# Synthesis, In Silico and In Vitro Assessment of New Quinazolinones as Anticancer Agents via Potential AKT Inhibition

**DOI:** 10.3390/molecules25204780

**Published:** 2020-10-18

**Authors:** Ahmed A. Noser, Mohamed El-Naggar, Thoria Donia, Aboubakr H. Abdelmonsef

**Affiliations:** 1 Organic Chemistry, Chemistry Department, Faculty of Science, Tanta University, Tanta 31527, Egypt; ahmed.nosir@science.tanta.edu.eg; 2 Chemistry Department, Faculty of Sciences, University of Sharjah, Sharjah 27272, UAE; melnagrr@sharjah.ac.ae; 3 Biochemistry Division, Chemistry Department, Faculty of Science, Tanta University, Tanta 31527, Egypt; thoria_donia@science.tanta.edu.eg; 4 Chemistry Department, Faculty of Science, South Valley University, Qena 83523, Egypt

**Keywords:** AKT1, cancer, quinazolinone, docking study, cytotoxic activity

## Abstract

A series of novel quinazolinone derivatives (**2**–**13**) was synthesized and examined for their cytotoxicity to HepG2, MCF-7, and Caco-2 in an MTT assay. Among these derivatives, compounds **4** and **9** exhibited significant cytotoxic activity against Caco-2, HepG2, and MCF-7 cancer cells. Compound **4** had more significant inhibitory effects than compound **9** on Caco-2, HepG2, and MCF-7 cell lines, with IC_50_ values of 23.31 ± 0.09, 53.29 ± 0.25, and 72.22 ± 0.14µM, respectively. The AKT pathway is one of human cancer’s most often deregulated signals. AKT is also overexpressed in human cancers such as glioma, lung, breast, ovarian, gastric, and pancreas. A molecular docking study was performed to analyze the inhibitory action of newly synthetic quinazolinone derivatives against *Homo sapiens* AKT1 protein. Molecular docking simulations were found to be in accordance with in vitro studies, and hence supported the biological activity. The results suggested that compounds **4** and **9** could be used as drug candidates for cancer therapy via its potential inhibition of AKT1 as described by docking study.

## 1. Introduction

Cancer is one of the most widespread illnesses in the world [1]. In 2030, the number of new cancer diagnoses is expected to be 21 million worldwide annually, with 17 million deaths because of cancer every year and 75 million people living with cancer diagnoses [2]. Due to the resistance to current cancer drugs and a lack of selectivity in tumor cells, the chemical design has become increasingly sophisticated over the years [3,4,5]. Targeted cancer therapies are designed to improve efficacy and selectivity by interfering with specific molecular targets and preventing the growth, development, and spread of cancer [6]. The importance of PKB/AKT protein kinase for cellular survival has been investigated in various cell types and animal systems and in response to several stress factors. The serine-threonine kinase AKT, also known as PKB, is a proto-oncogenic key player in cell proliferation, apoptosis, metabolism of glucose, and cell migration processes [7,8]. AKT activated by phosphorylation, which includes the binding of PI3K-phosphorylated phosphoinositides (PI) called PIP3 AKT pleckstrin homology domain and subsequent translocation to the plasma membrane and phosphorylation at two phosphorylation sites Thr308 and Ser473 by PDK1 and PDK2, respectively, resulting in its activation in tumor cells, so it was found that direct degradation of AKT deregulated AKT activity and promoted an apoptotic process [9,10,11]. Three AKT isoforms have been identified in the human genome (AKT1, AKT2, and AKT3) with a highly conserved pleckstrin homology domain. Under physiological conditions, AKT1 and AKT2 are ubiquitously expressed, whereas AKT3 expression is restricted, predominantly to heart, kidney, brain, testis, lung, and skeletal muscle [12].

The inhibition of AKT has therefore been regarded as a promising therapeutic approach in oncology for several years, and significant efforts have been made to find new effective and selective anti-cancer drugs for AKT [6,13,14,15,16].

The quinazolinone moiety is founded in many natural products and pharmaceutical drugs. The quinazolinone analogues have widespread applications ranging from biomedical science to material science [17], such as antioxidant [18,19], antidiabetic [20], anti-inflammatory [21], antibacterial [22], anticoagulant [23], antifibrotic [24,25], antiproliferative [26], anticonvulsant [27], and antituberculosis [28]. Moreover, the quinazolinone moiety is considered to be a major class of promising structural scaffold with PI3K inhibition [29]. Prompted by the aforementioned findings, and in attempt to develop potent anticancer agents targeting AKT protein, a new series of novel quinazolinone derivatives (**2–13**) was synthesized and investigated for their cytotoxic activity on cancer cell lines, i.e., HepG2 (human liver cancer), MCF-7 (human breast cancer), and Caco-2 (human colon cancer). As a part of our work, computer-based docking studies were performed, and absorption, distribution, metabolism, excretion and toxicity (ADMET) properties, as well as the Structure Activity Relationship (SAR) of the compounds, were determined to investigate their binding mode of interactions with the active site of the target AKT1.

In this study, the synthesis and in silico and in vitro evaluation of a series of quinazoline analogues as potential anticancer agents targeting AKT1 protein is described, as it is hyperactivated in many cancers.

## 2. Results and Discussion

### 2.1. Chemistry of the Synthesized Compounds

**Scheme 1:**
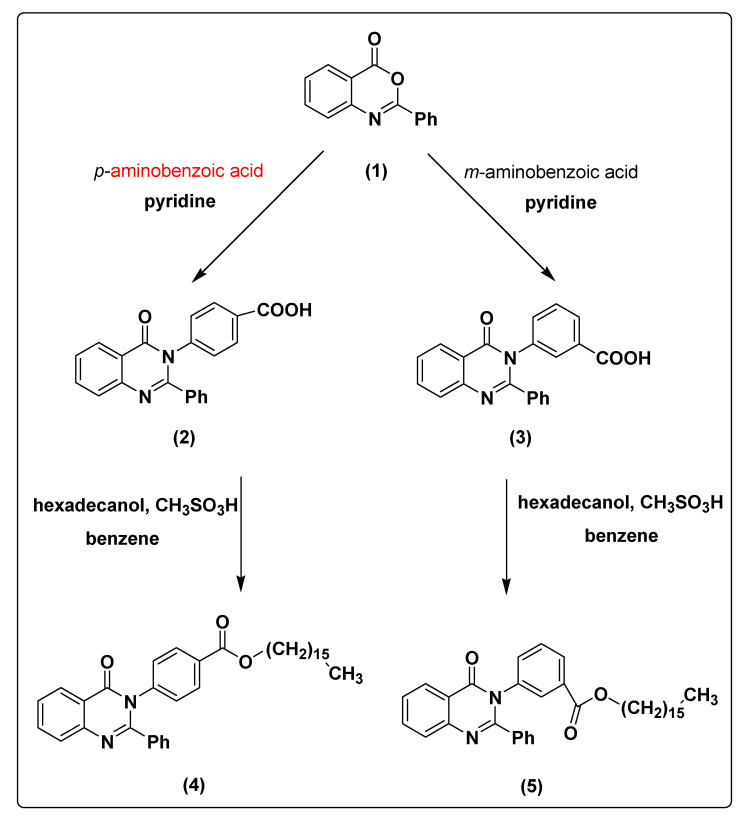
The synthesis pathway of compounds **2–5** from compound **1**.

The 2-phenyl-*4H*-benzo[*d*][1,3]oxazin-4-one (**1**) was synthesized and characterized as previously described in the literature [30]. In the present study, 4-(4-oxo-2-phenyl quinazolin-3(*4H*)-yl)benzoic acid (**2**) and 3-(4-oxo-2-phenylquinazolin-3(*4H*)-yl)benzoic acid (**3**) were obtained successfully by refluxing compound **1** with *p*-amino benzoic acid and *m*-amino benzoic acid respectively in the presence of pyridine, to give high yields of 88% and 87% respectively. Further chemical modification of compounds **2** and **3** with hexadecanol in the presence of methanesulfonic acid and benzene as solvent under reflux conditions using dean stark trap lead to formation of hexadecyl 4-(4-oxo-2-phenylquinazolin-3(*4H*)-yl)benzoate (**4**) and hexadecyl 3-(4-oxo-2-phenyl quinazolin-3(*4H*)-yl)benzoate (**5**), respectively, as shown in Scheme 1, with 90% and 88% yield. The structures of compounds **2–5** were elucidated via NMR spectroscopy, FT-IR, and elemental analysis as shown in Supplementary Materials.

**Scheme 2:**
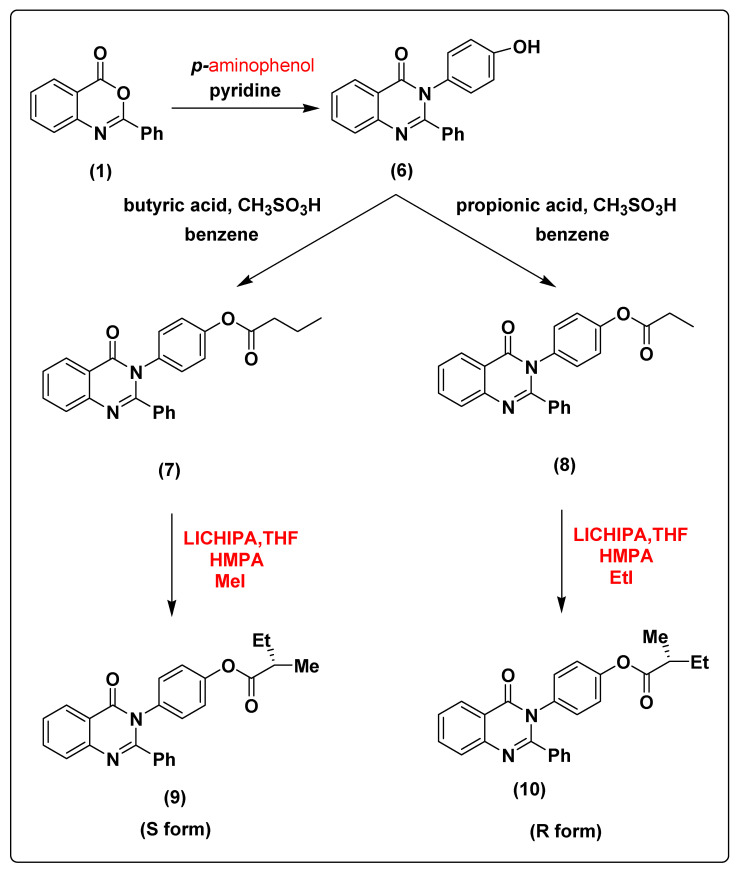
The synthesis pathway of compounds **6–10** from compound **1**.

The treatment of compound **1** with *p*-amino phenol leads to the formation of 3-(4-hydroxyphenyl)-2-phenylquinazolin-4(*3H*)-one (**6**) with 91% yield. The chemical modification of compound **6** with butyric acid and propionic acid in the presence of methanesulfonic acid and benzene as solvent under reflux conditions using dean stark trap leads to the formation of 4-(4-oxo-2-phenylquinazolin-3(*4H*)-yl)phenyl butyrate (**7**) and 4-(4-oxo-2-phenylquinazolin-3(*4H*)-yl)phenyl propionate (**8**) with 89% and 88% yield, respectively. The deprotonation of compound **7** using lithium cyclohexylisopropylamine (LICHIPA) at −96 °C followed by alkylation using methyl iodide in the presence of tetramethylethylenediamine (TMEDA) as catalyst lead to formation of (S)-4-(4-oxo-2-phenylquinazolin-3(*4H*)-yl)phenyl 2-methylbutanoate (**9**) with 90% yield. While the alkylation of compound **8** lead to the formation of (R)-4-(4-oxo-2-phenylquinazolin-3(*4H*)-yl)phenyl 2-methylbutanoate (**10**) with 90% yield (the change of the configuration occur according to the principle of order of addition, as represented in Scheme 2. The structures of compounds **6**, **7**, **8**, **9** and **10** were characterized via NMR spectroscopy, FT-IR and elemental analysis as shown in Supplementary Materials.

Formation of 3-hydroxy-4-(4-oxo-2-phenylquinazolin-3(*4H*)-yl)naphthalene-1-sulfonic acid (**11**) with 91% yield was achieved by treatment of compound **1** with 4-amino-3-hydroxynaphthalene-1-sulfonic acid.

**Scheme 3:**
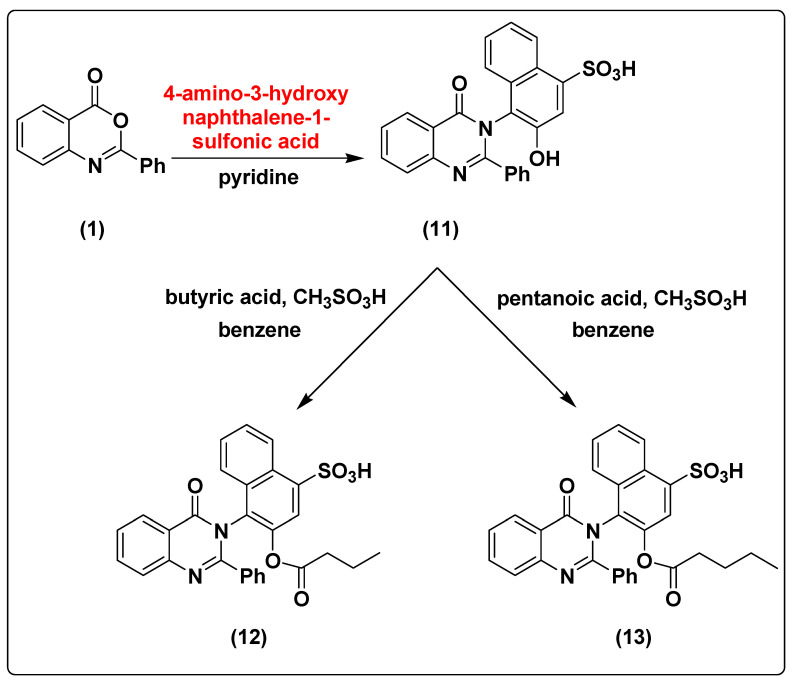
The synthesis pathway of compounds **11–13** from compound **1**.

The chemical modification of compound **11** with butyric acid and pentanoic acid in the presence of methanesulfonic acid and benzene as solvent under reflux conditions using dean stark trap with the same conditions mentioned above for the synthesis of compounds **7** and **8** afforded the formation of 4-(4-oxo-2-phenylquinazolin-3(*4H*)-yl)3-(butyryloxy)naphthalene-1-sulfonic acid (**12**) and 4-(4-oxo-2-phenylquinazolin-3(*4H*)-yl)3-(pentanoxy)naphthalene-1-sulfonic acid (**13**) with 88% and 87% yield, respectively, as shown in Scheme 3. The structures of compounds **11, 12,** and **13** were characterized using NMR spectroscopy, FT-IR and elemental analysis as represented in Supplementary Materials.

### 2.2. In Silico Docking Study

AKT pathway plays an important role in multiple cell signaling mechanisms implicated in cell metabolism, growth and division. Therefore, AKT1 has been selected as a promising target in cancer treatment [13,14,15,16]. Compound **2–13** exhibited significant cytotoxic activities against Caco-2, HepG2, and MCF-7 cancer cells, as represented in In Vitro Activity section. Based on these results, in silico docking technique was executed to identify the potential inhibitors with high efficiency against human AKT1 protein.

#### 2.2.1. The Crystal Structure and Active Site of the Target

The crystallographic structure of molecular target AKT1 obtained from the PDB database (PDB ID: 5WBL) was used for docking studies. Computational prediction tools declared that TYR46, ARG59, PRO176, GLN178, TYR180, TRP191, PHE193, LYS196, and ASP204 are the binding pockets of the target.

#### 2.2.2. Molecular Docking Analysis

**Figure 1:**
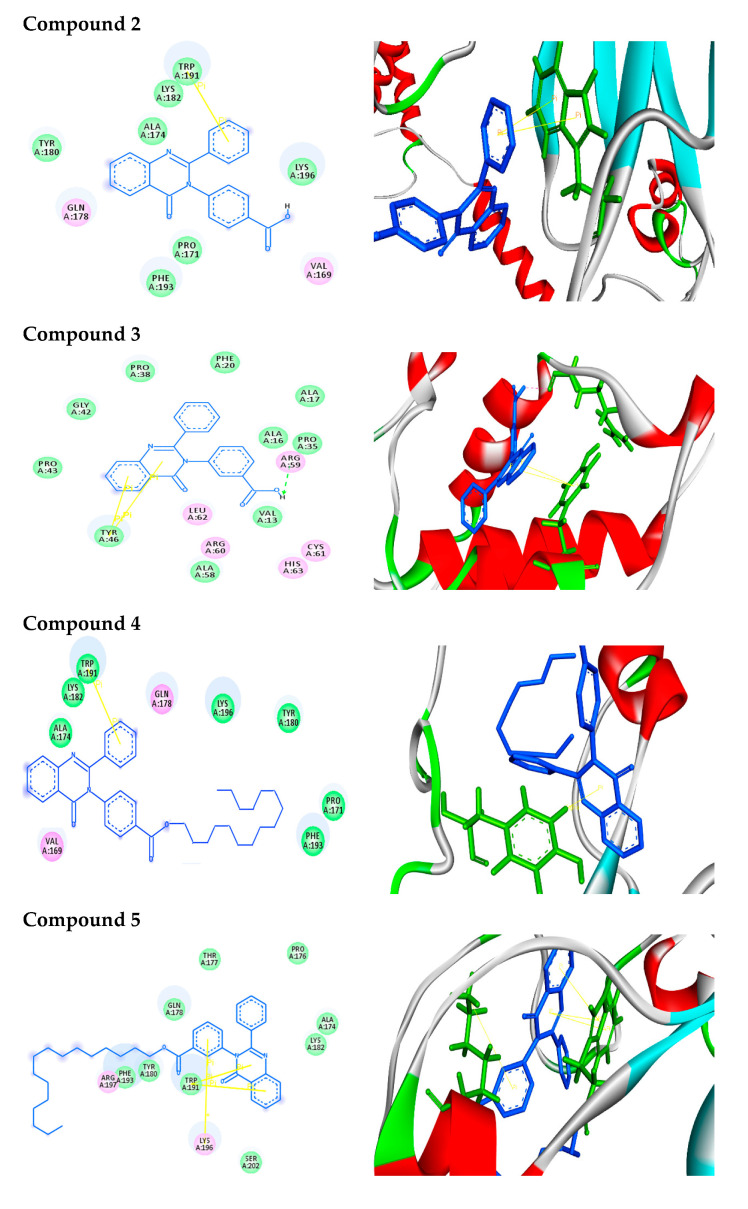

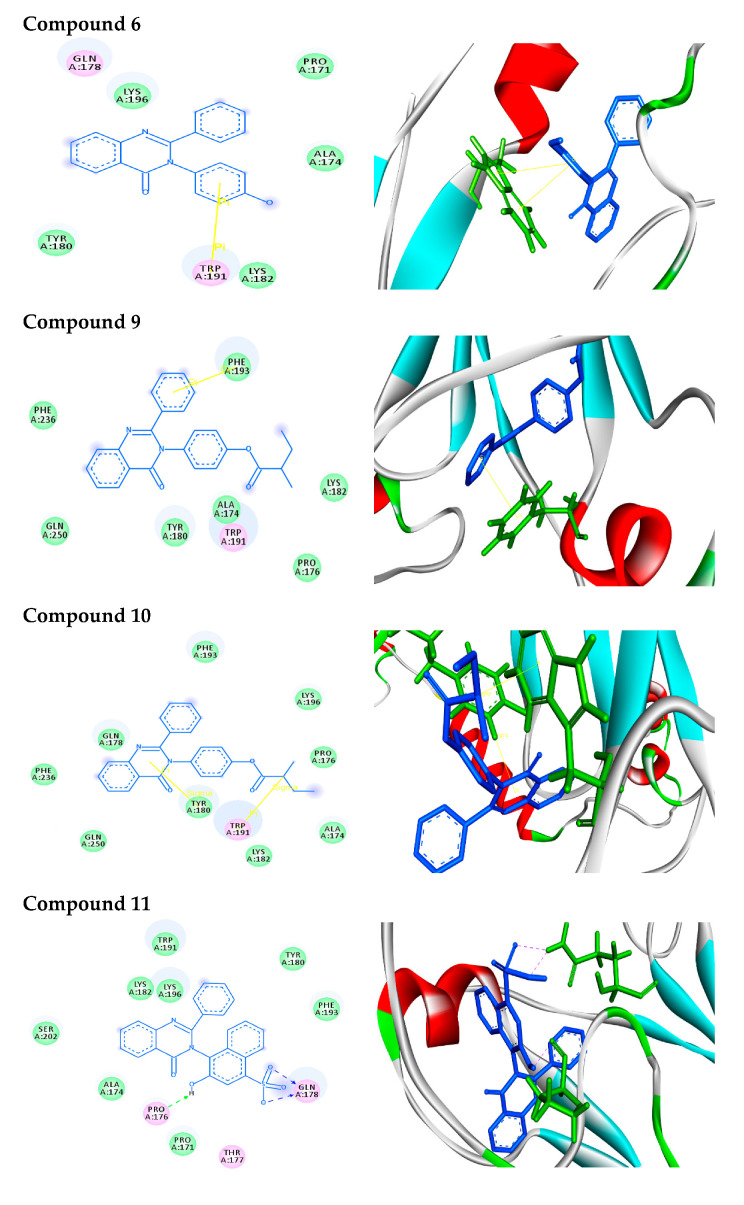

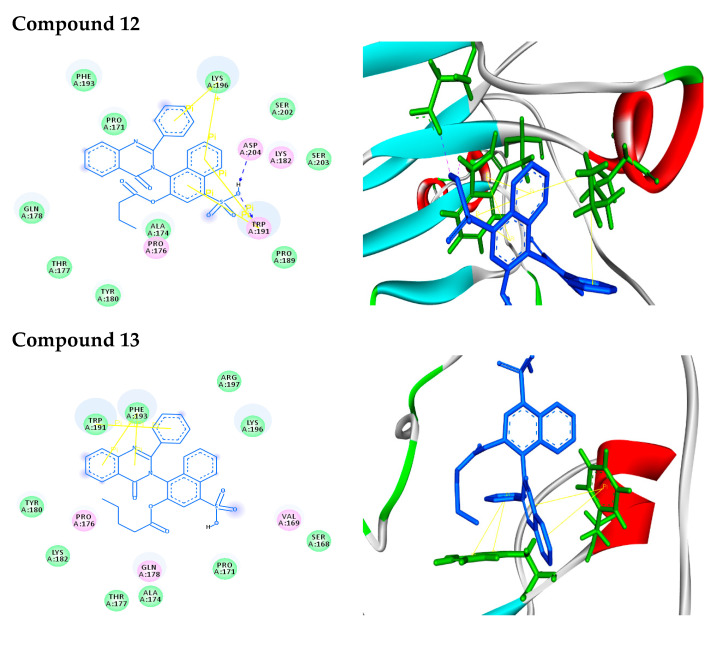
Binding modes/interactions of the synthetic compounds with active sites of AKT1 protein. (Left side) 2D representations demonstrating the molecular interactions between compounds and the active site region of AKT1 protein. The amino acid residues are shown in three-letter code, H-bonds are in pink doted lines, and π-interactions are in yellow lines. (Right side) 3D representations demonstrating the molecular interactions between compounds and the active site region of AKT1 protein. The compounds are represented by blue stick models, while the active site regions are shown by green stick models. H-bond contacts are shown in pink dotted lines, while π-stacking is shown in yellow lines.

For in silico docking, the approach was performed using the PyRx virtual screening 3D tool. In screening against AKT1, the synthesized compounds were docked to a three-dimensional model of the target protein. Nine conformers are considered for each ligand–protein complex and the most energetically favorable binding mode is chosen to identify the best-docked compound against human AKT1 protein. The docking study exhibited nice fitting of the new synthetic compounds into the active site of the target, as tabulated in Table 1. The quinazoline derivatives have significant interaction poses with the modeled AKT1 through hydrogen bonds, π–π, π–cation, and π–σ interactions. Greater values of negative binding free energy ΔG_b_ (reported in kcal/mol) indicate better matching between the ligand molecule and target [31,32,33,34]. The reference compound doxorubicin had the lowest binding free energy (ΔG_b_ = −7.6 kcal/mol) and exhibited two π–π stacking with TRP191. Compound **2** had a binding free energy ΔG_b_ = −7.9 kcal/mol and exhibited π–π stacking with TRP191 at distances of 4.07 and 3.90 A°, respectively. Tryptophan (TRP) contains two phenyl and pyrrole rings involved in forming two π–π interactions with phenyl ring of compound **2**. Compound **3** interacts with protein at ARG59 and TYR46 through one hydrogen bond (O–H–O) plus two π–π interactions with distances of 2.33, 4.04 and 5.10 A°, respectively. Tyrosine (TYR) contains a phenyl ring involved in forming two π–π interactions with phenyl and pyrimidine rings of compound **3**. Compound **4**, with the highest binding free energy (ΔG_b_ = −10.2 kcal/mol), interacts with protein at TYR180 forming one π–σ interaction at a distance of 2.84 A°. This is due to the interaction between the pyrimidine ring of the compound and the aromatic side chain of Tryptophan. Compound **5** possesses π–π and π–cation interactions with the target protein through TRP191 and LYS196. This is due to lysine (LYS) containing a positively charged ε-amino group that is involved in forming π–cation interaction with phenyl moiety in the compound. In addition, compound **6** forms two π–π interactions with the target through TRP191 at the distances of 4.41 and 3.94 A°, respectively. Compound **9** (S-isomer), with a binding free energy ΔG_b_ = –9.8 kcal/mol, showed π–π interaction with the target protein through PHE193. The results showed that phenylalanine (PHE) contains an aromatic ring that is involved in forming π–π interactions with the phenyl moiety of the compound. Meanwhile, compound **10** (R-isomer) with a dock score of -7.7 kcal/mol exhibited two π–σ interactions through TRP191 and TYR180. In addition, compound **11** showed three hydrogen bonds with GLN178 and PRO176 at distances of 2.10, 2.09 and 2.07 A°, respectively. Compound **12** showed rich network interactions, such as hydrogen bonds, π–π and π–cation interactions, with the target through TRP191, ASP204 and LYS196. Finally, compound **13** showed π–π interaction with TRP191 and PHE193. Figure 1 represents 2D (Left side) and 3D (Right side) docking interactions between molecules **2–13** and the active site of AKT1 protein.

#### 2.2.3. Docking Simulation

In 2D docking simulations, the amino acid residues are shown in three-letter code, H-bonds are in pink doted lines, and π-interactions are in yellow lines. Meanwhile, in 3D docking simulations, the binding residues of AKT1 protein are shown in green colored stick models and the ligands in blue one. The hydrogen bonds are represented by pink dotted lines, and π-interactions are shown by yellow lines. Hetero moieties like pyrimidine are observed to be a common pharmacophore group that interacts with the functional residues of the cancer target protein AKT1 through various interactions like hydrogen bonds and π-stacking, as shown in Table 2.

#### 2.2.4. ADMET Property Evaluation

As a part of our study, the in silico absorption, distribution, metabolic, excretion (ADME) and toxicity (T) of the newly synthesized quinazoline compounds were identified using the admetSAR tool, as shown in Table 2. Interestingly, all the newly synthesized compounds had good BBB^+^ values, which describe the ability of the compounds to cross the blood–brain barrier; these values were in the acceptable range. Additionally, the values show that the compounds can be absorbed by the human intestines and are non-carcinogenic. The results show that these compounds show better inhibition properties against AKT1 protein. Drug-likeness parameters of compounds were calculated using Mol inspiration software, as summarized in Table 3. The results show interesting values for the compounds, which obey Lipinski’s rule, whereas all the compounds have topological surface areas in the acceptable range. Furthermore, the numbers of H-bond acceptors and donors in the tested compounds are in an acceptable range. Finally, the compounds possess high numbers of rotatable bonds. The bioavailability radar gives an overview of the drug-likeness of molecule **9** as an example (see Supplementary Materials). The region in pink color indicates the range for each property. The boiled-egg plot between WLOGP and TPSA is used to predict gastrointestinal and brain penetration of the selected compound **9**, as shown in Supplementary Materials. The plot shows that the probability of a good BBB crossing is high. From all these results, we can conclude that all molecules exhibit good absorption and distribution within the body. These molecules can be considered potent antagonists against human AKT1 protein and can be used as anti-cancer agents.

### 2.3. In Vitro Activity

**Figure 2:**
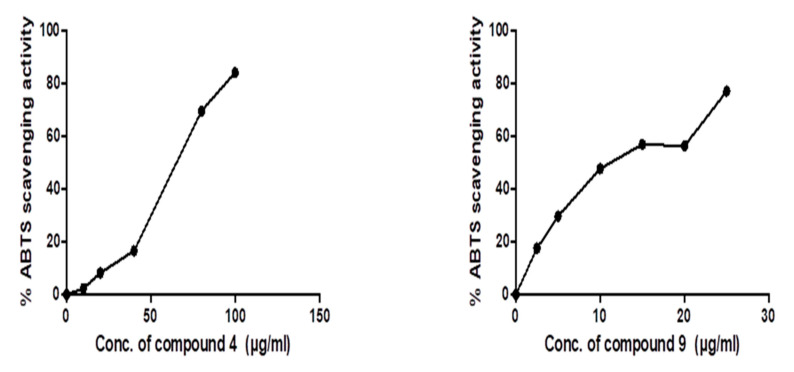
ABTS radical scavenging antioxidant activity of compounds **4** and **9**. The IC_50_ values of each drug are expressed as mean ± SE of three independent experiments performed in triplets calculated and plotted using Graphpad Prism software 6 (San Diego, CA, USA).

It was found, as shown in Figure 2, that the 50% ABTS scavenging activities of compounds **4** and **9** are 62.3 ± 0.09 and 18 ± 1.2, respectively.

**Figure 3:**
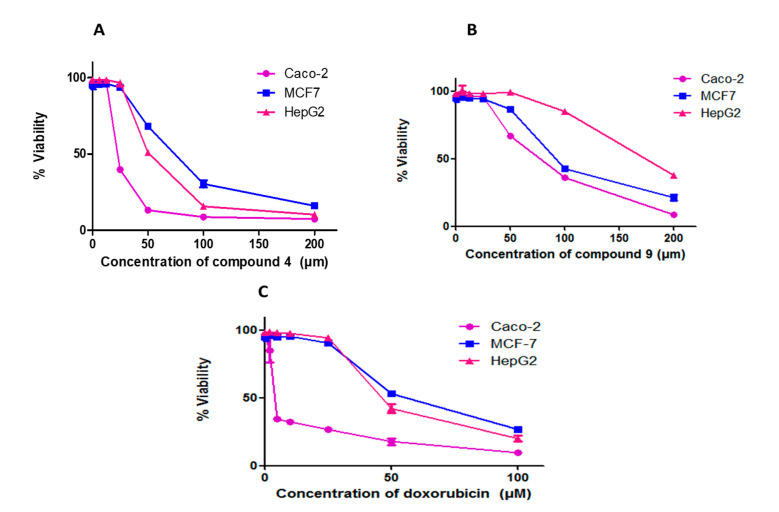
**Compounds 4 and 9** and doxorubicin inhibit proliferation in different cancer cell lines. Cells were treated with various concentrations of each compound for 48 h and cell viability was plotted against drug concentration to calculate IC_50._ The IC_50_ values of each drug are expressed as mean ± SE of three independent experiments performed in triplets calculated and plotted using Graphpad Prism software 6 (San Diego, CA).

In the research for new anticancer agents, the most common screening methods are screening tests against a panel of different cancer cell lines. In this study, (3-(4,5-dimethylthiazol-2-yl)-2,5-diphenyltetrazolium bromide (MTT) assay was carried out to determine the cytotoxic effects of the compounds on HepG2, MCF-7, and Caco-2 cancer cell lines (Figure 3 and Table 4). Compounds **4** and **9** exhibited significant cytotoxic activity against Caco-2, HepG2, and MCF-7 cancer cells. Compound **4** had more significant inhibitory effects than compound **9** on Caco-2, HepG2, and MCF-7 cell lines, with IC_50_ values of 23.31 ± 0.09, 53.29 ± 0.25, and 72.22 ± 0.14 µM, respectively. This compound was also as effective as doxorubicin (IC_50_ = 49.38 ± 0.15) on HepG2 cells. The IC_50_ values of compound **9** against HepG2, MCF-7, and Caco-2 cell lines were 171.4 ± 0.12, 96.58 ± 0.17 and 73.87 ± 0.13, respectively, which is less than doxorubicin (49.38 ± 0.15, 58.1 ± 0.07 and 5.7 ± 0.12, respectively). Introducing the substituents on phenyl ring affected the activity of the compounds, in addition, the position of the substituents on the phenyl ring affected the biological activity of the compound [35,36]. As can be seen in compound **4**, the substituent at the para position increased anticancer activity against the selected cancer cells than meta-position in compound **5**; additionally, the configuration of the compound affected its biological activity, as it was found that the S configuration in compound **9** was better than the R configuration in compound **10**.

### 2.4. Structure Activity Relationship (SAR)

In connection with the activity values found with the structural moieties of the newly synthetic compounds, it was declared that compounds **4** and **9** possessed the greatest activity. The cytotoxic activity of both compounds **4** and **9** against Caco-2, HepG2, and MCF-7 cancer cells was further investigated by the in silico molecular docking technique. Compound **4**, with the highest binding free energy (ΔGb = −10.2 kcal/mol), interacted with the target due to the interaction between the pyrimidine ring of the compound and the aromatic side chain of Tryptophan. Moreover, compound **9** (S-isomer), with a binding free energy ΔGb = –9.8 kcal/mol, showed π–π interaction with the target protein through PHE193. Compound **4** had more significant inhibitory effects than compound **9** on Caco-2, HepG2, and MCF-7 cell lines, with IC_50_ values of 23.31 ± 0.09, 53.29 ± 0.25, and 72.22 ± 0.14 µM, respectively. This compound was also as effective as doxorubicin (IC50 = 49.38 ± 0.15) on HepG2 cells. The IC_50_ values of compound **9** against HepG2, MCF-7, and Caco-2 cell lines were 171.4 ± 0.12, 96.58 ± 0.17 and 73.87 ± 0.13, respectively, which is less than doxorubicin (49.38 ± 0.15, 58.1 ± 0.07 and 5.7 ± 0.12, respectively). Introducing substituents on the phenyl ring affected the activity of the compounds; additionally, the position of the substituents on the phenyl ring affected the biological activity of the compound. As represented in compound **4**, the substituent at the para position increased anticancer activity against the selected cancer cells than meta-position in compound **5**; additionally, the configuration of the compound affected its biological activity, as it as found that the S configuration in compound **9** was better than the R configuration in compound **10**.

## 3. Materials and Methods

### 3.1. Chemistry

#### 3.1.1. General Information

Anthranilic acid, benzoyl chloride, p-aminobenzoic acid, m-aminobenzoic acid, p-aminophenol, cyclohexylisopropylamine (CHIPA), *N,N,N′,N′*-tetramethylethylenediamine (TMEDA), n-butyllithium solution 2.5 M in hexane (n-BuLi), hexadecanol, methyliodide (MeI), ethyliodide (EtI), propionic acid, butyric acid, pentanoic acid, tetrahydrofuran (THF), methanesulfonic acid (CH_3_SO_3_H), pyridine and benzene were purchased from Sigma-Aldrich Chemical Co. (St. Louis, MO, USA).

Reactions were monitored by TLC performed on precoated plates Merck Kieselgel 60 F254 (EMD Millipore corporation, Billerica, MA, USA). Infrared spectra were recorded at Tanta University by central laboratory using a Perkin Elmer 1420 spectrophotometer (Waltham, MA, USA), and the spectra were carried out by using KBr disc technique, the samples were dried in oven then mounted on a sample holder with a large cavity. Melting points were determined in degrees centigrade by the open capillary method using Gallenkamp melting point and were reported uncorrected.

The elemental analyses of compounds were performed at the micro analytical center, Cairo University using Perkin-Elmer 240 CHN Elemental analyzer, ^1^H NMR and ^13^C NMR spectra were collected at resonance frequencies of 400 MHz at Kafr El-sheikh university. NMR spectra were performed on a Bruker AMC instrument (Bruker Biosciences Corporation, Billerica, MA, USA) operating at 400 MHz using dimethyl sulfoxide (DMSO) as a solvent and tetramethylsilane as an internal standard. The chemical shifts for ^1^H NMR are reported in ppm from tetramethylsilane (0 ppm) or referenced to the solvent (DMSO-d_6_, δ2,50). Chemical shifts (δ) for ^13^C NMR spectra refer to the signals for residual deuterated solvents (DMSO-d_6_, 37.5). Multiplicities are reported by the following abbreviations: s (singlet), d (doublet), t (triplet), m (multiplet).

#### 3.1.2. Synthesis of 2-phenyl-4*H*-benzo[*d*][1,3]oxazin-4-one (**1**)

Compound **1** was prepared as described by Tiwary [30] with 86% Yield.

#### 3.1.3. Synthesis of 4-(4-oxo-2-phenylquinazolin-3(4*H*)-yl)benzoic acid (**2**)

In a 50 mL two-neck round-bottom flask a mixture of compound **1** (2.23 g, 10 mmol) and para amino benzoic acid (1.64 g, 12 mmol) in pyridine (30 mL) was refluxed for 6 h (TLC control) then poured into ice/water. The resulting solid was filtered, washed with water, and dried. Yield: 88%; mp: 270 °C; ^1^H NMR (400 MHz, DMSO-d_6_) δ (ppm): 7.21–8.11 (m, 13H, Ar-H), 11 (s, 1H, COOH); ^13^C NMR (400 MHz, DMSO-d_6_) δ (ppm): 169.3, 126, 131, 139, 122, 165, 161, 121, 129, 127.5, 177.7, 122.6, 151.7, 126.4; IR (KBr) ν: 3330 (OH), 3063 (Ar-H), 1715 (CO); Anal. Calcd for C_21_H_14_N_2_O_3_ (342.35): C, 73.68%; H, 4.12%; O, 14.02%; N, 8.18%. Found: C, 73.36%; H, 4.04%; N, 8.09%.

#### 3.1.4. Synthesis of 3-(4-oxo-2-phenylquinazolin-3(4*H*)-yl)benzoic acid (**3**)

In a 50 mL two-neck round-bottom flask a mixture of compound **1** (2.23 g, 10 mmol) and meta amino benzoic acid (1.64 g, 12 mmol) in pyridine (30 mL) was refluxed for 6 h (TLC control) then poured into ice/water. The resulting solid was filtered, washed with water, and dried. Yield: 87%; mp: 256 °C; ^1^H NMR (400 MHz, DMSO-d_6_) δ (ppm): 7.21–8.51 (m, 13H, Ar-H), 11 (s, 1H, COOH); ^13^C NMR (400 MHz, DMSO-d_6_) δ (ppm): 169.3, 130.7, 120, 132.9, 127, 128.7, 128.8, 128.9, 129, 126, 126.1, 164, 161, 121, 127.7, 133.5, 122.5, 151.5, 126.3, 130.2.

#### 3.1.5. Synthesis of hexadecyl 4-(4-oxo-2-phenylquinazolin-3(4*H*)-yl)benzoate (**4**)

In a 50 mL two-neck round-bottom flask a mixture of compound **2** (3.42 g, 10 mmol), hexadecanol (2.42 g, 10 mmol) and 1 mL of methanesulphonic acid in benzene (50 mL) was refluxed for 8 h under N_2_-gas using dean stark trap (TLC control) then the product was cooled and neutralized by 5% sodium carbonate. The resulting solid was filtered, washed with water, and dried. Yield: 90%; ^1^H NMR (400 MHz, DMSO-d_6_) δ (ppm): 7.08–8.21 (m, 13H, Ar-H), 0.96 (t, 3H, CH_3_), 1.23–1.75 (m, 28H, CH_2_), 4.11 (t, 2H, CH_2_); ^13^C NMR (400 MHz, DMSO-d_6_) δ (ppm): 166, 125.9, 130.2, 121.7, 137.3, 164.2, 161, 121, 128.9, 127.6, 133.7, 122.6, 151.5, 128.9, 126.3, 129, 130.4, 65, 29.2, 26, 29.6, 29.9, 32, 22.9, 14.3; IR (KBr) ν: 1675 (CO), 3063 (Ar-H), 2994 (aliph-H); Anal. Calcd for C_37_H_46_N_2_O_3_ (566.77): C, 78.41%; H, 8.18%; O, 8.47%; N, 4.94%. Found: C, 78.21%; H, 8.11%; N, 4.72%.

#### 3.1.6. Synthesis of hexadecyl 3-(4-oxo-2-phenyl quinazolin-3(4*H*)-yl)benzoate (**5**)

In a 50 mL two-neck round-bottom flask a mixture of compound **3** (3.42 g, 10 mmol), hexadecanol (2.42 g, 10 mmol) and 1 mL of methanesulphonic acid in benzene (50 mL) was refluxed for 8 h under N_2_-gas using dean stark trap (TLC control) then the product was cooled and neutralized by 5% sodium carbonate. The resulting solid was filtered, washed with water, and dried. Yield: 88%; ^1^H NMR (400 MHz, DMSO-d_6_) δ (ppm): 7.08–8.52 (m, 13H, Ar-H), 0.96 (t, 3H, CH_3_), 1.23–1.75 (m, 28H, CH_2_), 4.11 (t, 2H, CH_2_); ^13^C NMR (400 MHz, DMSO-d_6_) δ (ppm): 166, 130.2, 119.8, 132.9, 126, 129, 125.7, 164.2, 161, 121, 128.9, 127.6, 133.7, 122.6, 151.5, 128.9, 126.3, 130.4, 126.5, 65, 29.1, 26, 29.6, 29.9, 32, 22.9, 14.3.

#### 3.1.7. Synthesis of 3-(4-hydroxyphenyl)-2-phenylquinazolin-4(3*H*)-one (**6**)

In a 50 mL two-neck round-bottom flask a mixture of compound **1** (2.23 g, 10 mmol) and para amino phenol (1.30 g, 12 mmol) in pyridine (30 mL) was refluxed for 6 h (TLC control) then poured into ice/water. The resulting solid was filtered, washed with water, and dried. Yield: 91%; mp: 220 °C; ^1^H NMR (400 MHz, DMSO-d_6_) δ (ppm): 7.21–7.91 (m, 13H, Ar-H), 5 (s, 1H, OH); ^13^C NMR (400 MHz, DMSO-d_6_) δ (ppm): 164, 161, 121, 128.8, 127.6, 133.7, 122.6, 151.6, 128.9, 126.3, 129, 130.3, 125.6, 123, 116.3, 154.3; IR (KBr) ν: 3320 (OH), 3063 (Ar-H), 1735 (CO); Anal. Calcd for C_20_H_14_N_2_O_2_ (314.34): C, 76.42%; H, 4.49%; O, 10.18%; N, 8.91%. Found: C, 76.22%; H, 4.32%; N, 8.66%.

#### 3.1.8. Synthesis of 4-(4-oxo-2-phenylquinazolin-3(4*H*)-yl)phenyl butyrate (**7**)

In a 50 mL two-neck round-bottom flask a mixture of compound **6** (3.14 g, 10 mmol), butyric acid (0.91 g, 10 mmol) and 1 mL of methanesulphonic acid in benzene (50 mL) was refluxed for 8 h under N_2_-gas using dean stark trap (TLC control) then the product was cooled and neutralized by 5% sodium carbonate. The resulting solid was filtered, washed with water, and dried. Yield: 89%; mp: 230 °C; ^1^H NMR (400 MHz, DMSO-d_6_) δ (ppm): 7.08–8.21 (m, 13H, Ar-H), 0.96 (t, 3H, CH_3_), 1.66 (m, 2H, CH_2_), 2.44 (t, 2H, CH_2_); ^13^C NMR (400 MHz, DMSO-d_6_) δ (ppm): 127.6, 133.7, 122.6, 151.5, 121, 129, 164, 161, 129.8, 121.8, 147, 122, 172.5, 35.9, 18.5, 13.7, 128.9, 126.3, 129,130.2; IR (KBr) ν: 1675 (CO), 3063 (Ar-H), 2994 (aliph-H); Anal. Calcd for C_24_H_20_N_2_O_3_ (384.43): C, 74.98%; H, 5.24%; O, 12.49%; N, 7.29%. Found: C, 74.68%; H, 5.14%; N, 7.19%.

#### 3.1.9. Synthesis of 4-(4-oxo-2-phenylquinazolin-3(4*H*)-yl)phenyl propionate (**8**)

In a 50 mL two-neck round-bottom flask a mixture of compound **6** (3.14 g, 10 mmol), propionic acid (1.09 mL, 10 mmol) and 1 mL of methanesulphonic acid in benzene (50 mL) was refluxed for 8 h under N_2_-gas using dean stark trap (TLC control) then the product was cooled and neutralized by 5% sodium carbonate. The resulting solid was filtered, washed with water, and dried. Yield: 88%; mp: 260 °C; ^1^H NMR (400 MHz, DMSO-d_6_) δ (ppm): 7.08–8.21 (m, 13H, Ar-H), 1.11 (t, 3H, CH_3_), 2.14 (m, 2H, CH_2_); ^13^C NMR (400 MHz, DMSO-d_6_) δ (ppm): 172.3, 27.4, 9.5, 147, 121.8, 122, 129,8, 121.8, 164, 161, 121, 129, 127.6, 133.7, 122.6, 151.5, 128.9, 126.3, 130.5; IR (KBr) ν: 1675 (CO), 3063 (Ar-H), 2994 (aliph-H); Anal. Calcd for C_23_H_18_N_2_O_3_ (370.40): C, 74.58%; H, 4.90%; O, 12.96%; N, 7.56%. Found: C, 74.38%; H, 4.64%; N, 7.29%.

#### 3.1.10. Synthesis of (S)-4-(4-oxo-2-phenylquinazolin-3(4*H*)-yl)phenyl 2-methylbutanoate (**9**)

In a 100 mL two-neck round-bottom flask(3.84 g, 10 mmol) of compound **7** was swelled in 20 mL THF overnight, then (3.32 mL, 20 mmol) of CHIPA was added at −96 °C (using methanol and liquid nitrogen) as cooling bath, then (3 mL, 15 mmol) of *n*-BuLi was added drop wise then the reaction mixture was stirred for 1 h under N_2_-gas, then 1 mL of TMEDA was added as catalyst and finally (1.24 mL, 20 mmol) of methyl iodide was added drop wise at −40 °C, then the reaction mixture was stirred overnight. The resulting solid was filtered, washed with water, and dried [37,38]. Yield: 90%; ^1^H NMR (400 MHz, DMSO-d_6_) δ (ppm): 7.05–8.22 (m, 13H, Ar-H), 0.98 (t, 3H, CH_3_), 1.20 (d, 3H, CH_3_), 1.60 (m, 2H, CH_2_), 2.55 (m, 1H, CH); ^13^C NMR (400 MHz, DMSO-d_6_) δ (ppm): 127.6, 133.5, 122.5, 151.5, 121, 128.8, 164, 161, 128.7, 129.8, 122, 121.9, 147, 175.5, 41, 16.5, 126, 129, 130, 26.5, 12; IR (KBr) ν: 1725 (CO), 3069 (Ar-H), 2989 (aliph-H); Anal. Calcd for C_25_H_22_N_2_O_3_ (398.45): C, 75.36%; H, 5.57%; O, 12.05%; N, 7.03%. Found: C, 75.13%; H, 5.37%; N, 6.92%.

#### 3.1.11. Synthesis of (R)-4-(4-oxo-2-phenylquinazolin-3(*4H*)-yl)phenyl 2-methylbutanoate (**10**)

In a 100 mL two-neck round-bottom flask(3.70 g, 10 mmol) of compound **8** was swelled in 20 mL THF overnight, then (3.32 mL, 20 mmol) of CHIPA was added at -96 °C (using methanol and liquid nitrogen) as cooling bath, then (3 mL, 15 mmol) of *n*-BuLi was added drop wise then the reaction mixture was stirred for 1h under N_2_-gas, then 1 mL of TMEDA was added as catalyst and finally (1.60 mL, 20 mmol) of ethyl iodide was added drop wise at -40 °C; then, the reaction mixture was stirred overnight. The resulting solid was filtered, washed with water, and dried. Yield: 90%.

#### 3.1.12. Synthesis of 3-hydroxy-4-(4-oxo-2-phenylquinazolin-3(4*H*)-yl)naphthalene-1-sulfonic acid (**11**)

In a 50 mL two-neck round-bottom flask a mixture of compound **1** (2.23 g, 10 mmol) and 4-amino-3-hydroxynaphthalene-1-sulfonic acid (2.87 g, 12 mmol) in pyridine (30 mL) was refluxed for 6 h (TLC control) then poured into ice/water. The resulting solid was filtered, washed with water, and dried. Yield: 91%; ^1^H NMR (400 MHz, DMSO-d_6_) δ (ppm): 7.21–7.95 (m, 14H, Ar-H), 2 (s, 1H, S-OH), 5 (s, 1H, C-OH); ^13^C NMR (400 MHz, DMSO-d_6_) δ (ppm): 133.4, 117.5, 135.3, 126.7, 126.4, 120, 125.8, 124.5, 127, 125.4, 164, 161, 121, 129, 127.5, 133.5, 122.5, 151.5, 128.7, 126.3, 129.2, 130.5; IR (KBr) ν: 3320 (OH), 3065 (Ar-H), 1735 (CO); Anal. Calcd for C_24_H_16_N_2_O_5_S (444.46): C, 64.86%; H, 3.63%; O, 18.00%; S, 7.21%. Found: C, 64.56%; H, 3.34%; S, 7.11%.

#### 3.1.13. Synthesis of 4-(4-oxo-2-phenylquinazolin-3(4*H*)-yl)3-(butyryloxy)naphthalene-1-sulfonic acid (**12**)

In a 50 mL two-neck round-bottom flask a mixture of compound **11** (4.44 g, 10 mmol), butyric acid (0.91 mL, 10 mmol) and 1 mL of methanesulphonic acid in benzene (50 mL) was refluxed for 8 h under N_2_-gas using dean stark trap (TLC control) then the product was cooled and neutralized by 5% sodium carbonate. The resulting solid was filtered, washed with water, and dried. Yield: 88%; ^1^H NMR (400 MHz, DMSO-d_6_) δ (ppm): 7.08–7.91 (m, 14H, Ar-H), 2 (s, 1H, S-OH), 0.96 (t, 3H, CH_3_), 1.55 (m, 2H, CH_2_), 2.36 (t, 2H, CH_2_); ^13^C NMR (400 MHz, DMSO-d_6_) δ (ppm): 132.9, 121, 130.5, 135.6, 125.5, 121.2, 125.9, 126.5, 127, 127.7, 164, 161, 121, 130, 127.5, 133.5, 122.5, 151.5, 128.7, 126.3, 130.5, 129, 172.5, 35.9, 18.5, 13.5.; IR (KBr) ν: 3345 (OH), 1675 (CO), 3063 (Ar-H), 2994 (aliph-H); Anal. Calcd for C_28_H_22_N_2_O_6_S (514.55): C, 65.36%; H, 4.31%; O, 18.66%; N, 5.44%; S, 6.23. Found: C, 65.16%; H, 4.21%; N, 4.14; S, 6.11%.

#### 3.1.14. Synthesis of 4-(4-oxo-2-phenylquinazolin-3(*4H*)-yl)3-(pentanoxy)naphthalene-1-sulfonic acid (**13**)

In a 50 mL two-neck round-bottom flask a mixture of compound **1**1 (4.44 g, 10 mmol), pentanoic acid (1.09 mL, 10 mmol) and 1 mL of methanesulphonic acid in benzene (50 mL) was refluxed for 8 h under N_2_-gas using dean stark trap (TLC control) then the product was cooled and neutralized by 5% sodium carbonate. The resulting solid was filtered, washed with water, and dried. Yield: 87%; ^1^H NMR (400 MHz, DMSO-d_6_) δ (ppm): 7.08–7.91 (m, 14H, Ar-H), 2 (s, 1H, OH), 0.96 (t, 3H, CH_3_), 1.33–1.55 (m, 4H, 2CH_2_), 2.36 (t, 2H, CH_2_); ^13^C NMR (400 MHz, DMSO-d_6_) δ (ppm): 133, 120.7, 130.5, 135.8, 125, 121, 125.5, 126.3, 127, 128, 164, 161, 121.2, 128.8, 127.5, 133.5, 122.5, 151.5, 128.9, 126, 129, 130.3, 172.5, 33.5, 27.5, 22.5, 14; IR (KBr) ν: 3375 (OH), 1695 (CO), 3083 (Ar-H), 2974 (aliph-H); Anal. Calcd for C_29_H_24_N_2_O_6_S (528.58): C, 65.90%; H, 4.58%; O, 18.16%; N, 5.30%; S, 6.07. Found: C, 65.56%; H, 4.41%; N, 5.14; S, 5.91%.

### 3.2. In Silico Study

A three-dimensional structure of human AKT1 protein was downloaded from the RSCB protein Data Bank [39]. An in-house database of ten quinazolinone compounds was created in SDF (Standard file format). All the compounds were energy minimized and used in the virtual screening study [40,41,42]. The binding pockets of the target were identified using 3DLigandSite and MetaPocket2.0 tools [43,44]. A grid is created around the binding pockets of AKT1 to perform screening. The docking was carried out by PyRx screening tool [45]. Finally, in silico pharmacokinetic and molecular properties of the synthesized quinazoline derivatives are predicted using various software’s such as admetSAR [46] http://lmmd.ecust.edu.cn/admetsar1/, Mol inspiration https://www.molin spiration.com/and SwissADME http://swissadme.ch/ web-based tools to select the compounds having optimum drug-likeness.

### 3.3. In Vitro ABTS Radical Scavenging Antioxidant and Anticancer Activities of Predicted Compounds

2,2 azino-bis3-ethylbenthiazoline-6-sulfonic acid (ABTS), ascorbic acid and DMSO were purchased from Sigma-Aldrich Chemical Co. USA. MTT solution was obtained from BIO BASIC CANADA INC.

#### 3.3.1. Evaluation of ABTS Radical Scavenging Activity

Total antioxidant activity was estimated according to the method described by Re et al. [47]. Briefly, 0.1 mL of different concentration of compound **4** (10–100 µg/mL) and 2.5–25 µg/mL for compound **9** was mixed with ABTS (pregenerated by adding 5ml of 4.9 mM potassium persulphate solution to 5 mL of a 14 mM ABTS solution and incubate 16h in dark). The mixture was shaken vigorously and allowed to stand in the dark at room temperature for 6 min. Absorbance of the resulting solution was monitored at 734 nm spectrophotometrically within 6 min. of reaction. The percentage of scavenged ABTS radical was calculated from the following equation:

ABTS scavenging % = (A_o_ − A_s_/A_o_) × 100 (1)

where A_o_ is the absorbance of the blank. A_s_ is the absorbance of sample and standard at 734 nm.

IC_50_, which denotes the amount (µg) of a sample in 1 mL solution required to reduce the initial concentration of ABTS radicals by 50%, was calculated using Graphpad Prism 5.

#### 3.3.2. Cell Culture

Three cancer cell lines namely HepG2 (human liver cancer), MCF-7 (human breast cancer) and Caco-2 (human colon cancer), were included in the study. Cell lines were cultured in Dulbecco’s modified Eagle’s medium (DMEM) supplemented with 10% fetal bovine serum and maintained in a 95% humidified incubator at 37 °C supplied with 5% CO_2_. All the reagents were purchased Gibco-BRL, USA.

#### 3.3.3. Determination of Compounds Cytotoxicity on Cells (MTT Protocol)

Cell proliferation (viability) was evaluated by MTT assay to establish IC_50_ (concentration to inhibit 50% of cells) according to the method of Denizot and Lang [48]. In brief, cells were seeded in 96-well plates to final count 1× 10^5^ cells/mL (3 × 10^4^/well) and incubated at 37 °C in 5% CO_2_ humidified incubator then left overnight for adhesion. The cells were treated by replacing the old media with another medium containing different concentration of investigated compounds (6.25–200 µM) and doxorubicin as reference drug, ranging from (6.25–200 µM) in triplicate for each concentration. The microplate was incubated in CO_2_ incubator for 48 h. At the end of treatment, the supernatant from each well was discarded and 20 μL of MTT (5 mg/mL) were added into each well for additional 4 h incubation at 37 °C. After that, the supernatant from each well was removed and the formazan crystals formed by viable cells were dissolved with DMSO (200 μL/well) with shaking (at highest speed) for 15 min at room temperature. The absorbance was read using Bio-RAD micro plate reader (Japan) at 570 nm. The OD measurements for control wells were considered to correspond 100% growth, their relative OD then calculated the percentage growth in other wells.

The percentage of viability was calculated as follow:

%Viability= Sample absorbance/Control absorbance × 100(2)

The potent compounds in docking studies were selected to study ABTS antioxidant activity as well as anticancer effect using MTT assay on three cancer cell lines.

## 4. Conclusions

In the present work, the synthesis and in silico and in vitro evaluation of a series of quinazoline analogues were described as potential anticancer agents targeting AKT1 protein. Among them, compounds **4** and **9** were the most effective anticancer agents on the tested cancer cell lines and the docking results were fundamentally in agreement with the biological data. According to the in vitro and in silico studies, compound **4** stands out as a promising orally bioavailable anticancer drug candidate for further in vivo study. The need for evaluation of their effect on AKT isoforms and downstream substrates is important to elucidate the molecular mechanisms. Furthermore, in vivo studies in cancer models are important for confirming its efficacy and additional mechanisms as anticancer agents. Additionally, toxicity to host cells is an important characteristic to assess the safety of drug candidates early in the drug discovery process. In summary, the results indicated that the quinazolinone derivatives could be used as potential inhibitors for cancer treatment via AKT1 inhibition.

## Figures and Tables

**Table 1:** The binding free energy ΔG_b_ (kcal/mol) of the newly synthetic compounds with AKT after molecular docking.

	Structure	Binding Energy (kcal/mol)	Docked Complex (Amino Acid-Ligand) Interactions	Distance (A°)
2	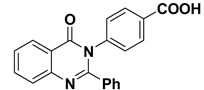	−7.9	**π–π interactions**	
			compound 2—TRP191	4.07
			compound 2—TRP191	3.90
3	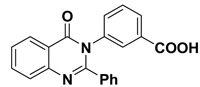	−9.3	**H-bonds**	
			compound 3—ARG59:O	2.33
			**π–π interactions**	
			compound 3—TYR46	4.04
			compound 3— TYR46	5.10
4	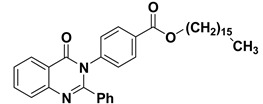	−10.2	**π–σ interactions**	
			compound 4—TYR180:HE1	2.84
5	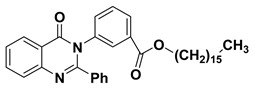	−8.5	**π–π interactions**	
			compound 5—TRP191	3.92
			compound 5—TRP191	3.91
			compound 5—TRP191	3.99
			compound 5—TRP191	4.51
			**π–cation interactions**	
			compound 5—LYS196:NZ	6.19
6	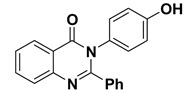	−8.0	**π–π interactions**	
			compound 6—TRP191	4.41
			compound 6—TRP191	3.94
9	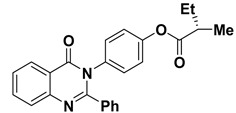	−9.8	**π–π interactions**	
			compound 9—PHE193	4.00
10	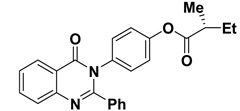	−7.7	**π–σ interactions**	
			compound 10—TRP191	3.65
			compound 10—TYR180:HE1	2.72
11	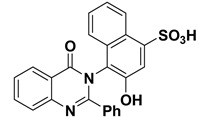	−8.9	**H-bonds**	
			compound 11—GLN178:HE22	2.10
			compound 11—GLN178:HE22	2.09
			compound 11—-PRO176:O	2.07
12	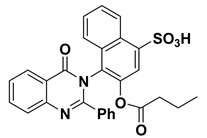	−9.2	**H-bonds**	
			compound 12—TRP191:HE1	2.48
			compound 12—ASP204:OD1	2.24
			**π–π interactions**	
			compound 12—TRP191	3.90
			compound 12— TRP191	3.99
			compound 12— TRP191	3.87
			compound 12— TRP191	5.10
			**π–cation interactions**	
			compound 12—LYS196:NZ	4.67
			compound 12—LYS196:NZ	5.24
13	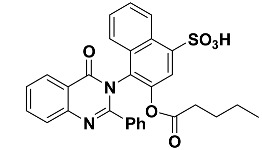	−9.1	**π–π interactions**	
			compound 13— TRP191	4.01
			compound 13— TRP191	4.41
			compound 13— PHE193	5.34
			compound 13— PHE193	4.80

All synthesized molecules with the best binding energy are represented with docking interactions in the table showing H-bonding, π–π, π–cation, and π–σ interactions.

**Table 2:** List of ADMET properties of the newly synthesized molecules.

	Molecular Weight (g/mol)	Blood–brain Barrier (BBB+)	Human Intestinal Absorption (HIA+)	Caco-2 Permeability (Caco-2)	AMES Toxicity	Carcinogenicity
2	342.35	0.958	0.969	0.619	Nontoxic	Non carcinogenic
3	342.35	0.958	0.969	0.619	Nontoxic	Non carcinogenic
4	566.79	0.979	0.995	0.531	Nontoxic	Non carcinogenic
5	566.79	0.979	0.995	0.531	Nontoxic	Non carcinogenic
6	314.34	0.975	0.997	0.548	Nontoxic	Non carcinogenic
9	398.46	0.977	0.997	0.600	Nontoxic	Non carcinogenic
10	398.46	0.977	0.997	0.600	Nontoxic	Non carcinogenic
11	444.47	0.615	0.859	0.586	Nontoxic	Non carcinogenic
12	514.56	0.670	0.840	0.601	Nontoxic	Non carcinogenic
13	528.59	0.692	0.856	0.596	Nontoxic	Non carcinogenic

The pharmacokinetic properties of the synthesized molecules are evaluated by admetSAR.

**Table 3:** Physicochemical properties of the synthesized compounds.

	TPSA (A^2^)	HBA	HBD	N rotatable	Volume (A^3^)
2	72.20	5	1	3	298.05
3	72.20	5	1	3	298.05
4	61.20	5	0	19	567.60
5	61.20	5	0	19	567.60
6	55.12	4	1	2	279.06
9	61.20	5	0	6	365.77
10	61.20	5	0	6	365.77
11	109.50	7	2	3	362.51
12	115.57	8	1	7	432.62
13	115.57	8	1	8	449.42

TPSA, topological polar surface area; HBA, number of hydrogen bond acceptors; HBD, number of hydrogen bond donors; N rotatable, number of rotatable bonds.

**Table 4:** IC_50_ of compound **4** and compound **9** as well as doxorubicin (as a reference drug) on different cancer cell lines.

IC50			
Compound	HepG2	MCF-7	Caco-2
4	53.29 ± 0.25	72.22 ± 0.14	23.31 ± 0.09
9	171.4 ± 0.12	96.58 ± 0.17	73.87 ± 0.13
Doxorubicin	49.38 ± 0.15	58.1 ± 0.07	5.7 ±1.2

Data are expressed as mean ± SE for three independent experiments using Graphpad Prism software 6 (San Diego, CA, USA).

## Supplementary Materials

The following are available online. ^1^H and ^13^C NMR spectra of the synthesized compound. The oral bioavailability radar of the molecule **9** and the Boiled-egg plot of the selected compound **9**.
