# Supplementary material for: Synthesis, In Silico and In Vitro Assessment of New Quinazolinones as Anticancer Agents via Potential AKT Inhibition
Source: Molecules. 2020 Oct 18;25(20):4780. doi: 10.3390/molecules25204780 (PMC7594071; doi:10.3390/molecules25204780)
Supplement: Supplementary file 1 [file molecules-25-04780-s001.pdf]

Supplementary Materials

Synthesis, *in silico* and *in vitro* assessment of new quinazolinones as anticancer agents via potential AKT inhibition

Ahmed A. Noser[1], Mohamed El-Naggar[2], Thoria Donia[3] and Aboubakr H. Abdelmonsef[4*]

[1] Organic Chemistry, Chemistry Department, Faculty of Science, Tanta University, Tanta 31527, Egypt
[2] Chemistry Department, Faculty of Sciences, University of Sharjah, 27272 Sharjah, UAE
[3] Biochemistry Division, Chemistry Department, Faculty of Science, Tanta University, Tanta 31527, Egypt
[4] Chemistry Department, Faculty of Science, South Valley University, Qena 83523, Egypt
* Correspondence: aboubakr.ahmed@sci.svu.edu.eg

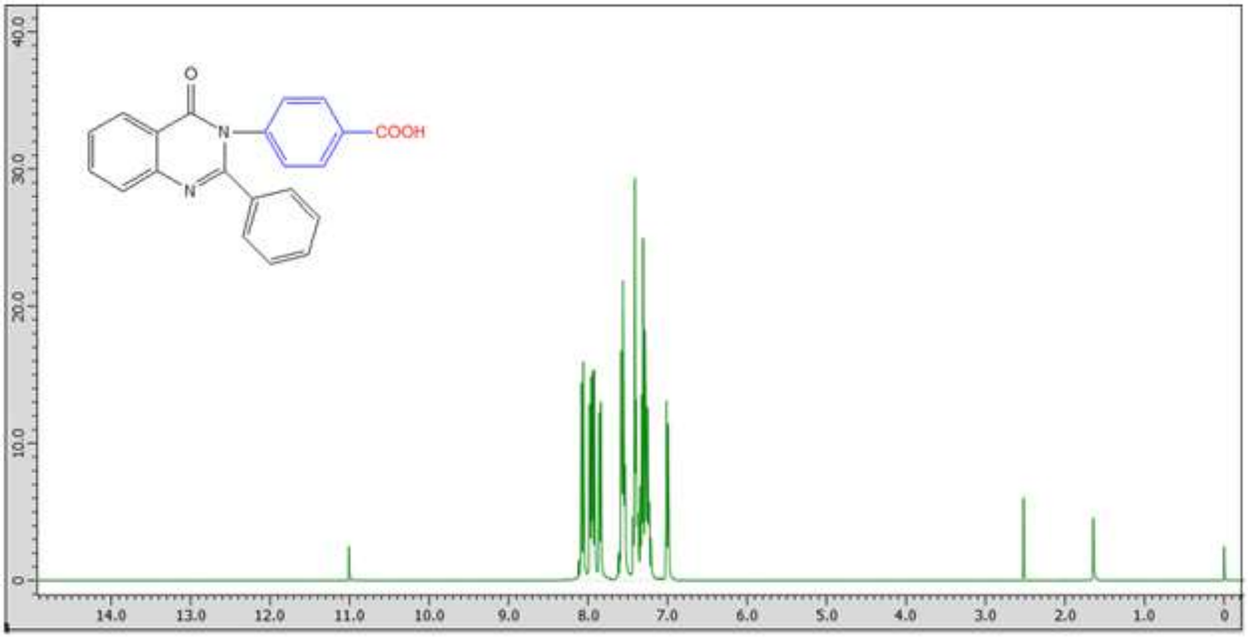

^{1}H-NMR spectrum of compound 2.

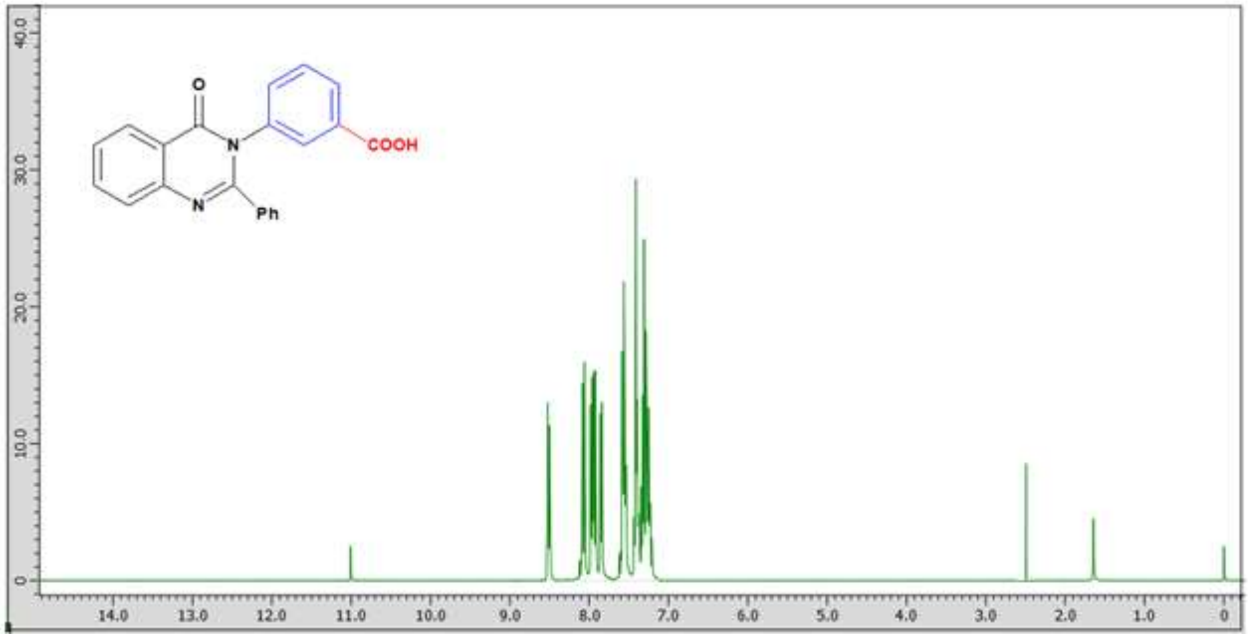

^{1}H-NMR spectrum of compound 3.

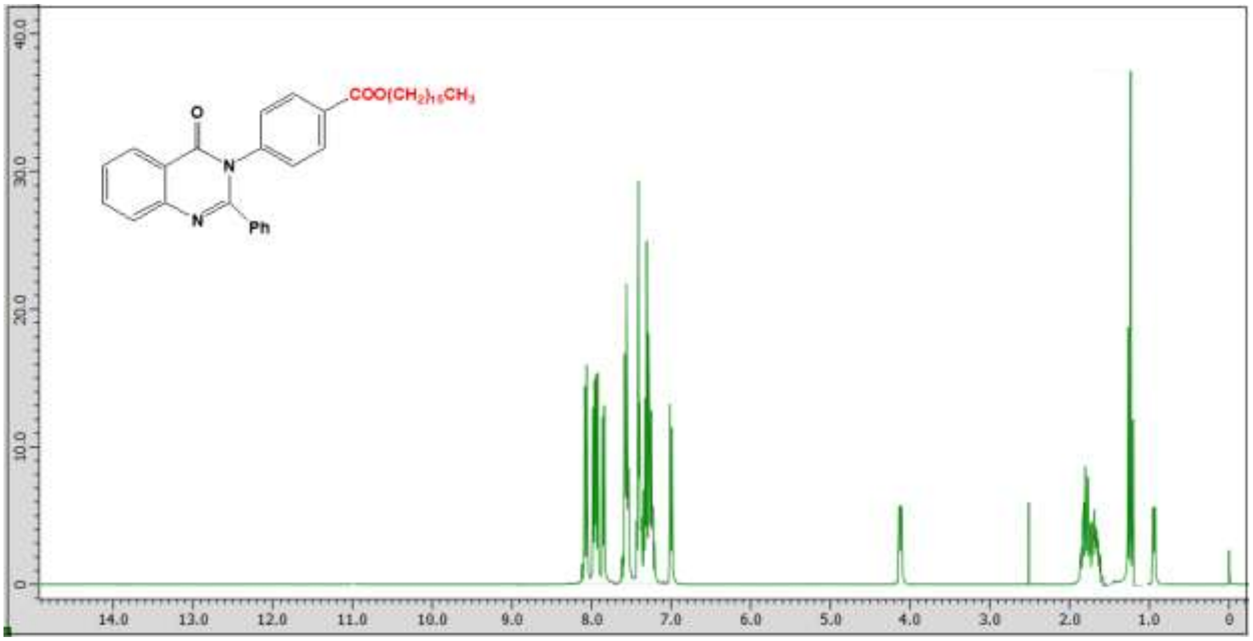

^{1}H-NMR spectrum of compound 4.

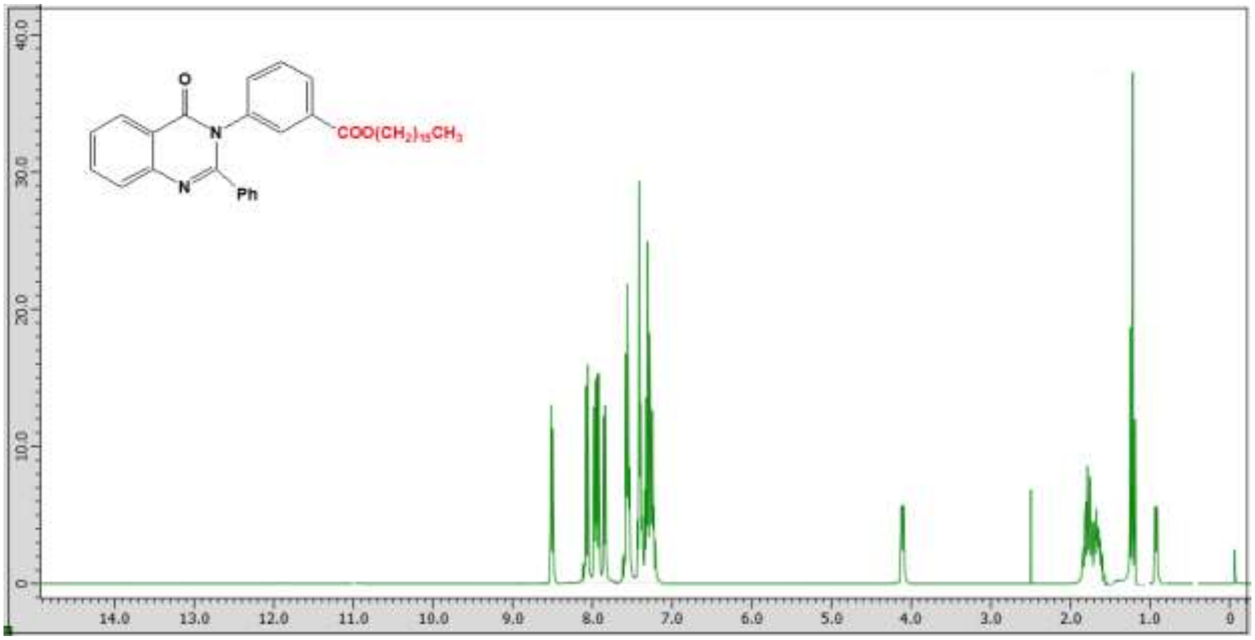

^{1}H-NMR spectrum of compound 5.

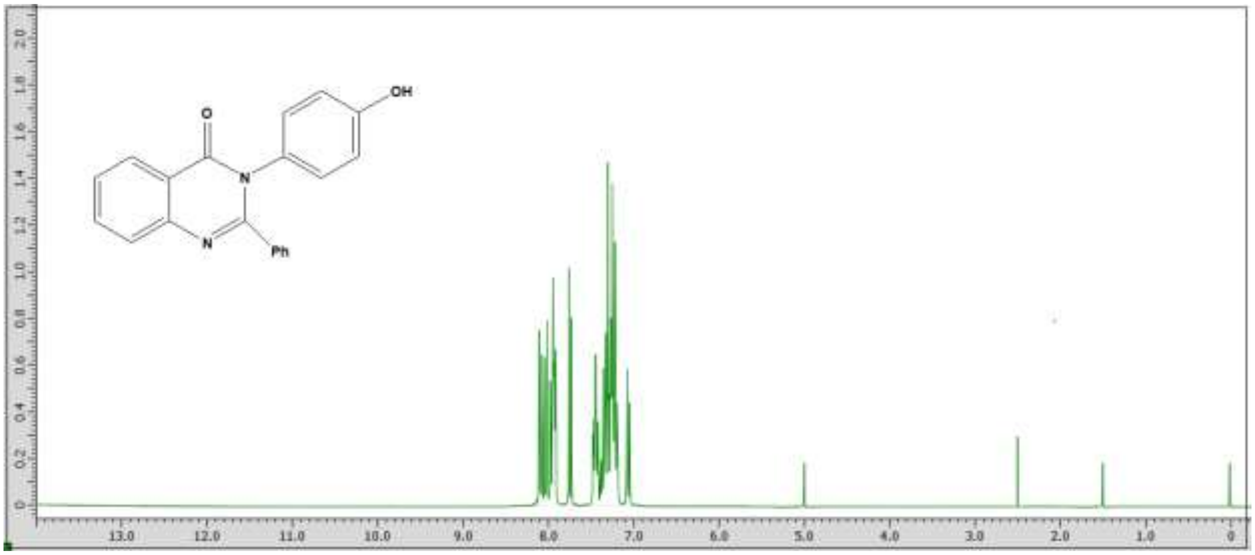

^{1}H-NMR spectrum of compound 6.

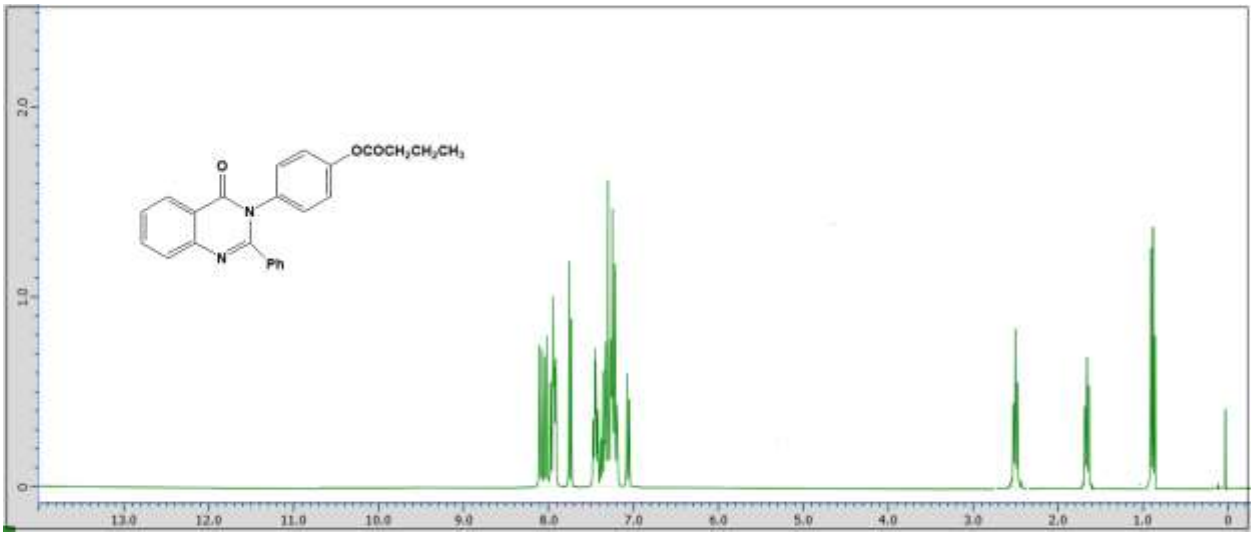

^{1}H-NMR spectrum of compound 7.

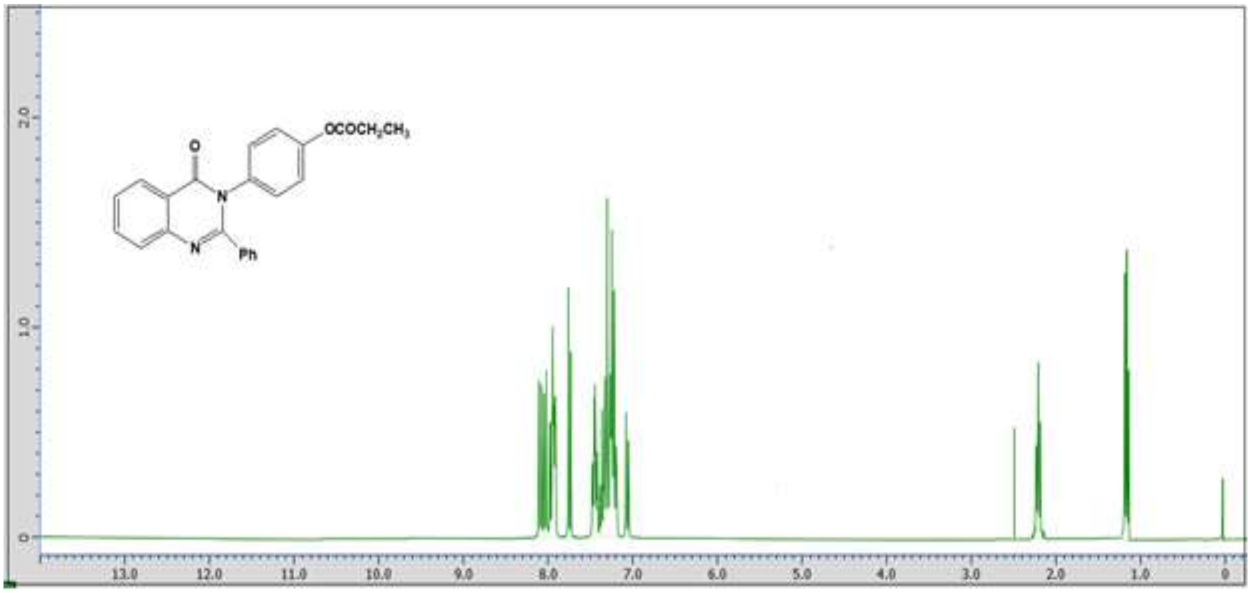

^{1}H-NMR spectrum of compound 8.

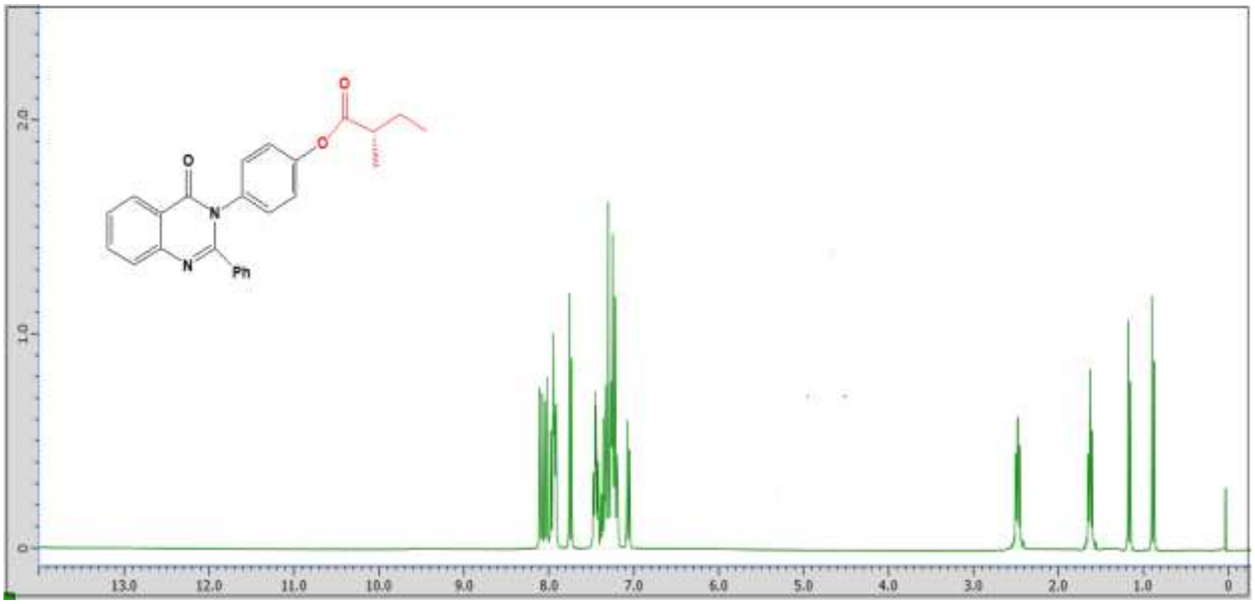

^{1}H-NMR spectrum of compound 10.

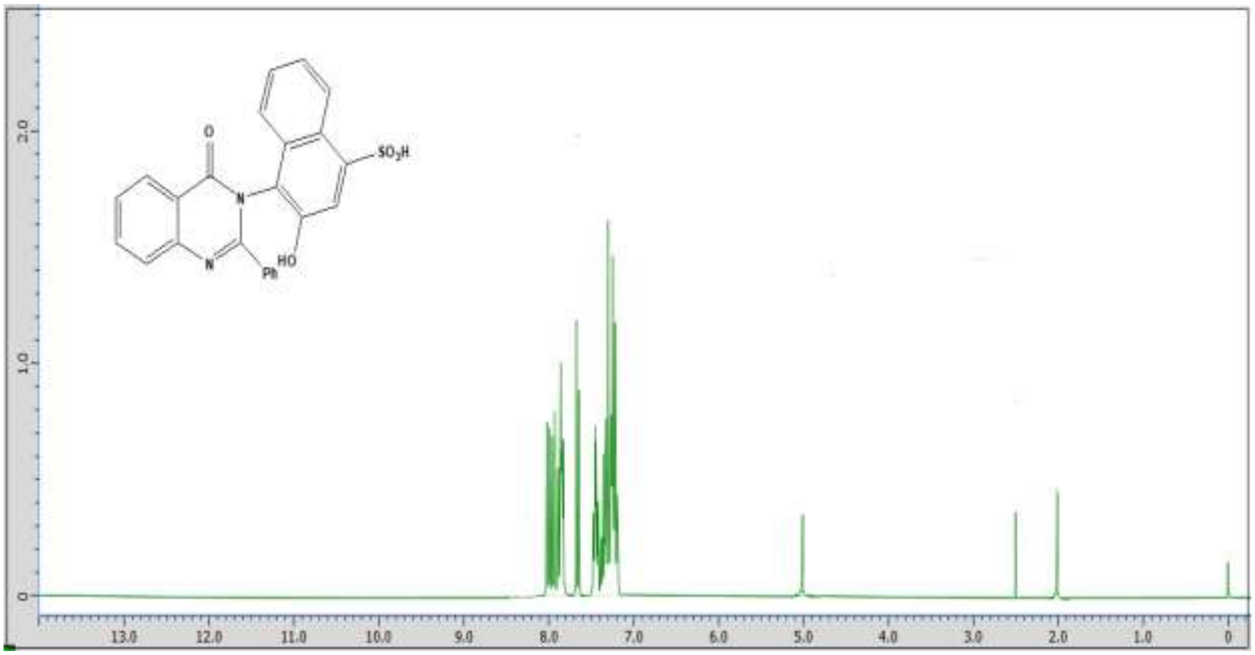

^{1}H-NMR spectrum of compound 11.

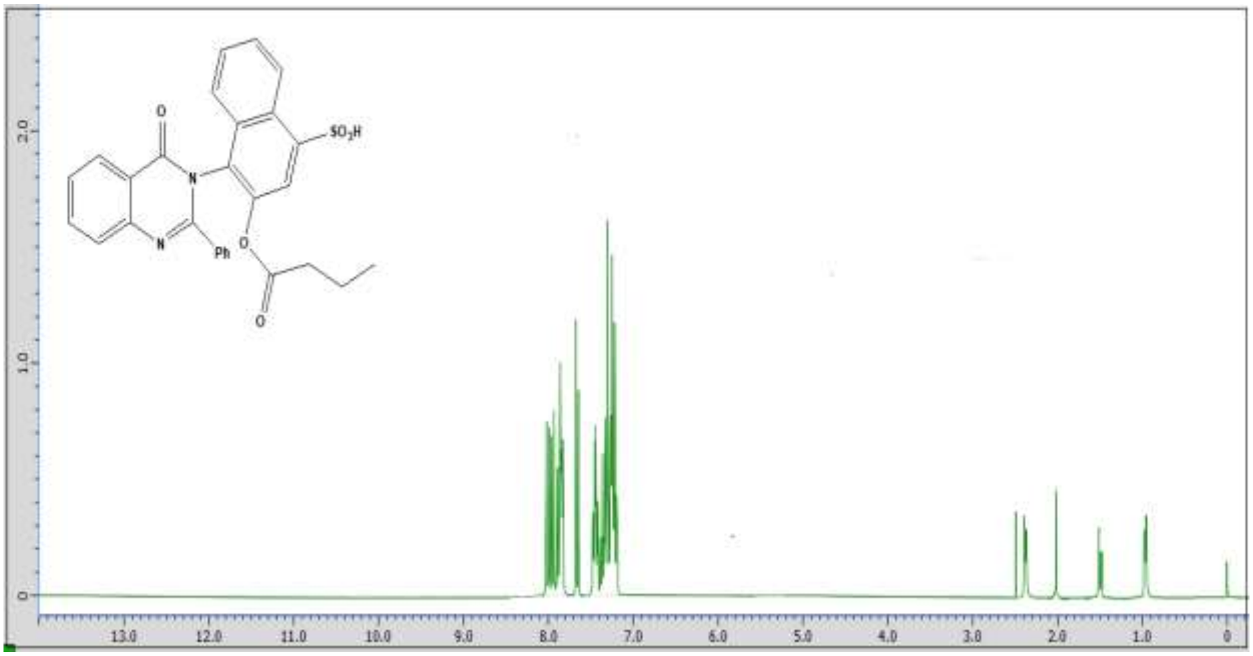

^{1}H-NMR spectrum of compound 12.

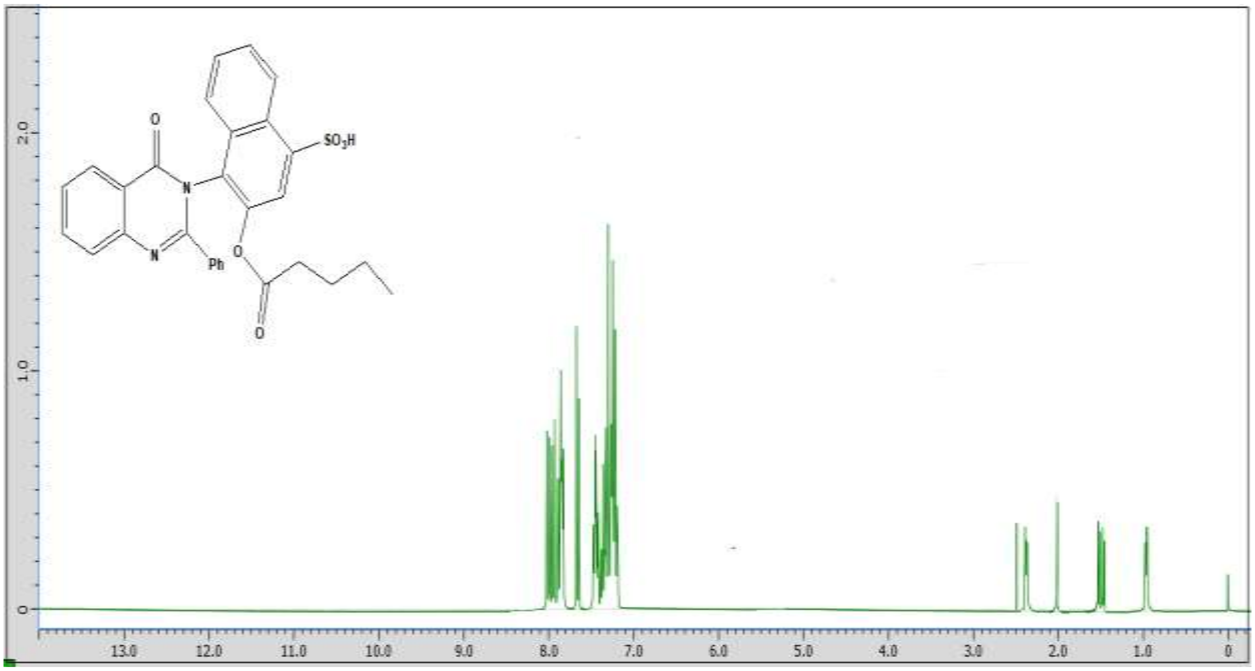

^{1}H-NMR spectrum of compound 13.

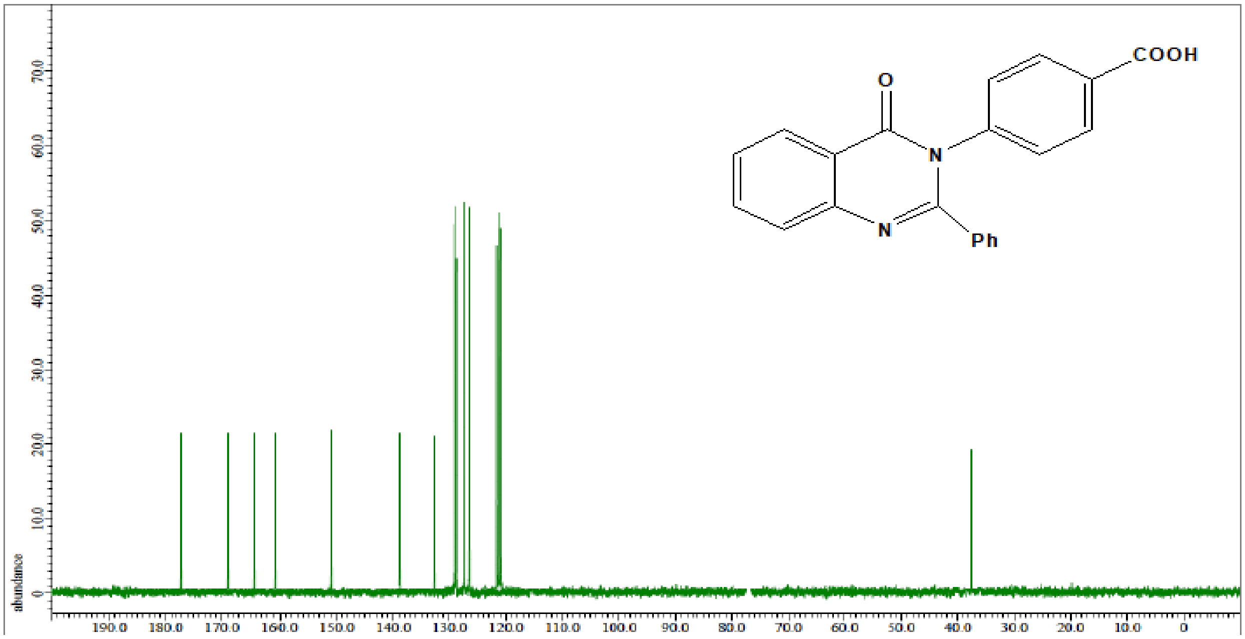

^{13}C-NMR spectrum of compound 2.

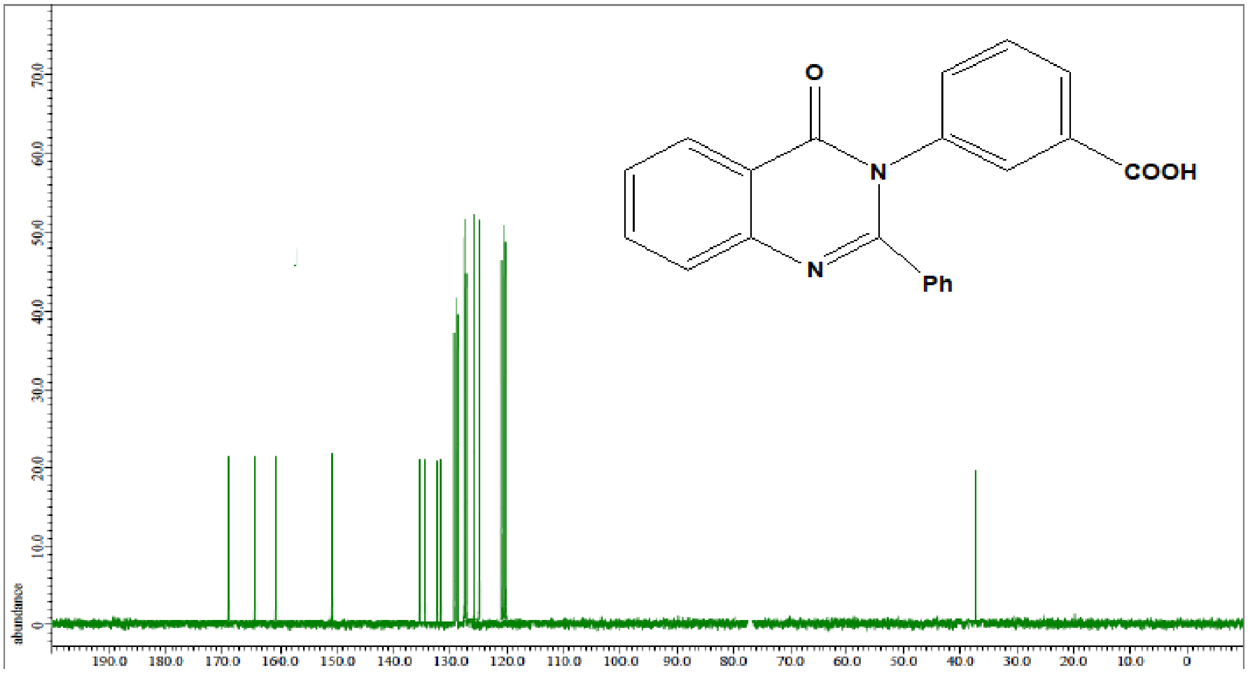

^{13}C-NMR spectrum of compound 3.

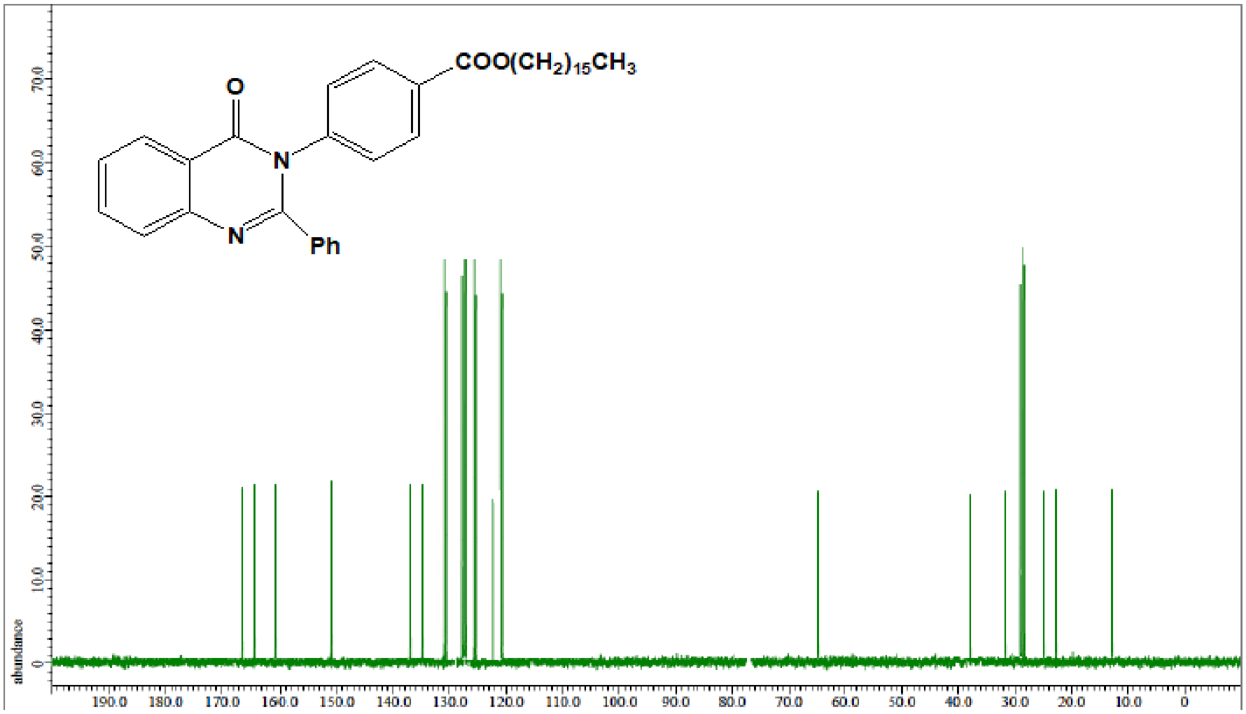

^{13}C-NMR spectrum of compound 4.

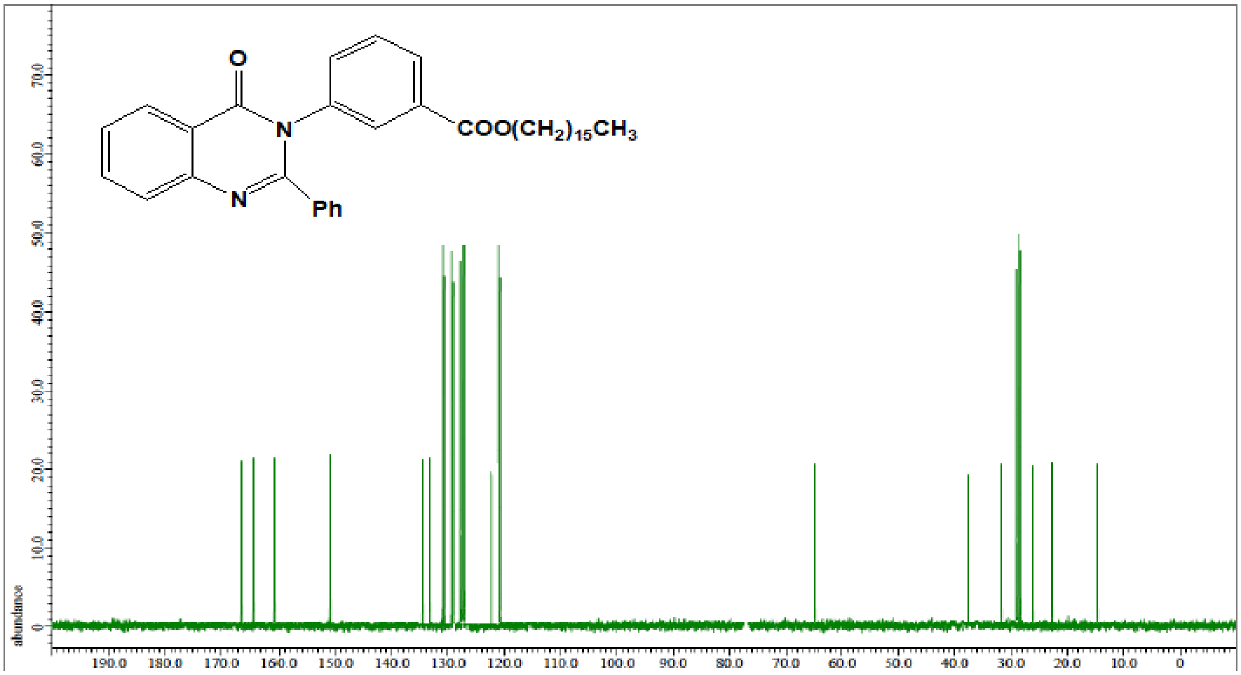

^{13}C-NMR spectrum of compound 5.

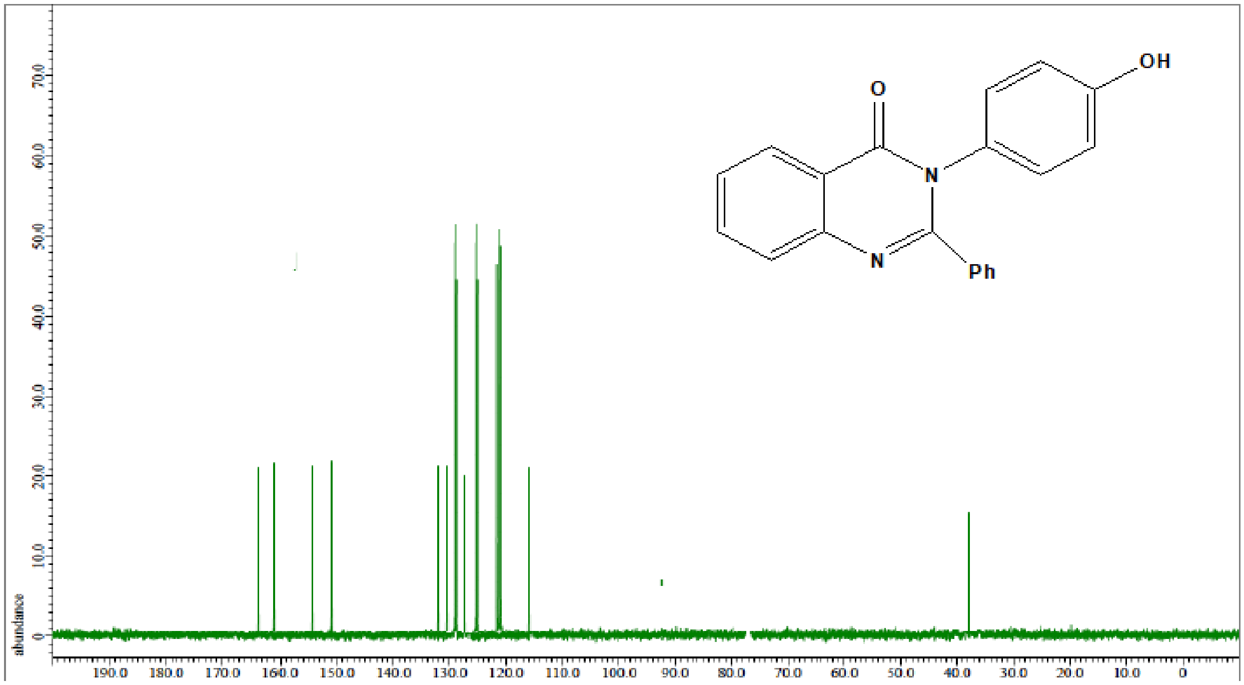

^{13}C-NMR spectrum of compound 6.

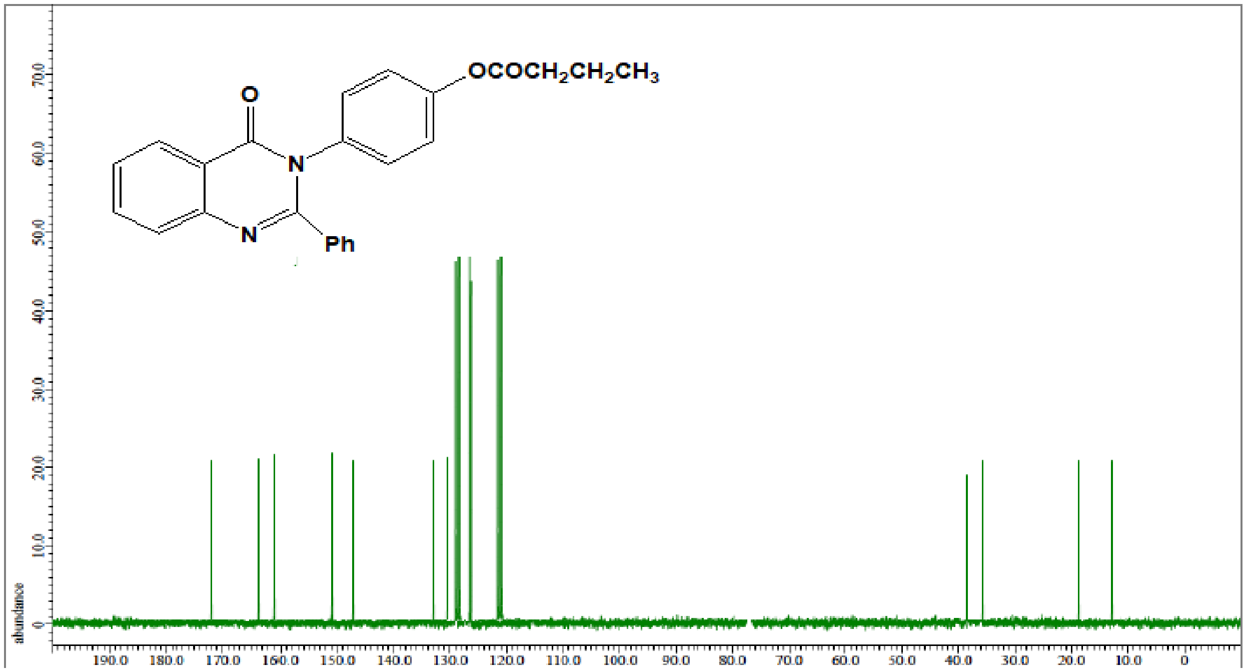

^{13}C-NMR spectrum of compound 7.

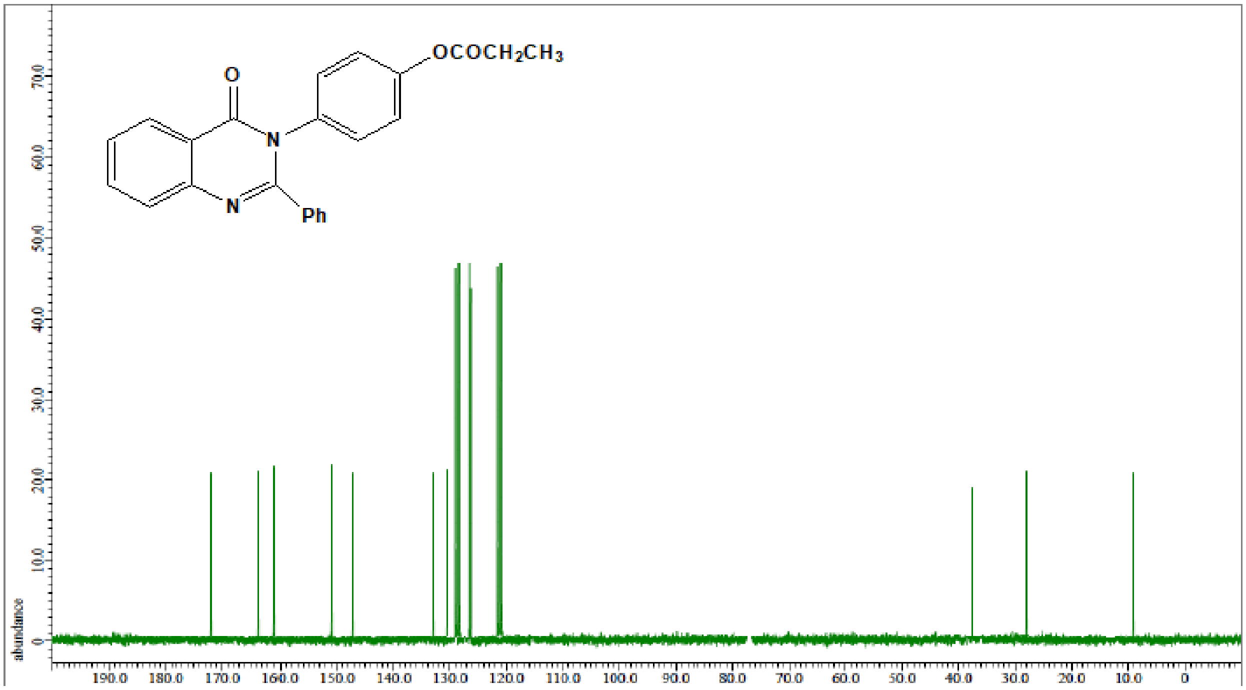

^{13}C-NMR spectrum of compound 8.

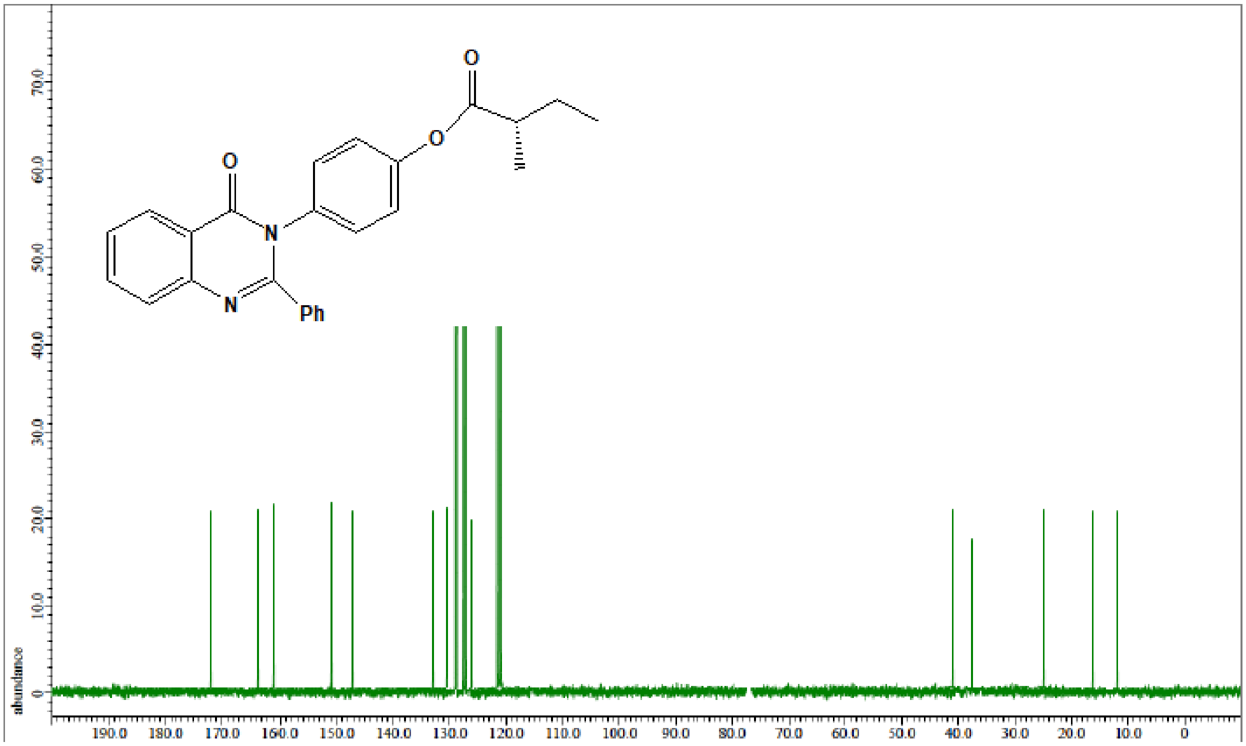

^{13}C-NMR spectrum of compound 10.

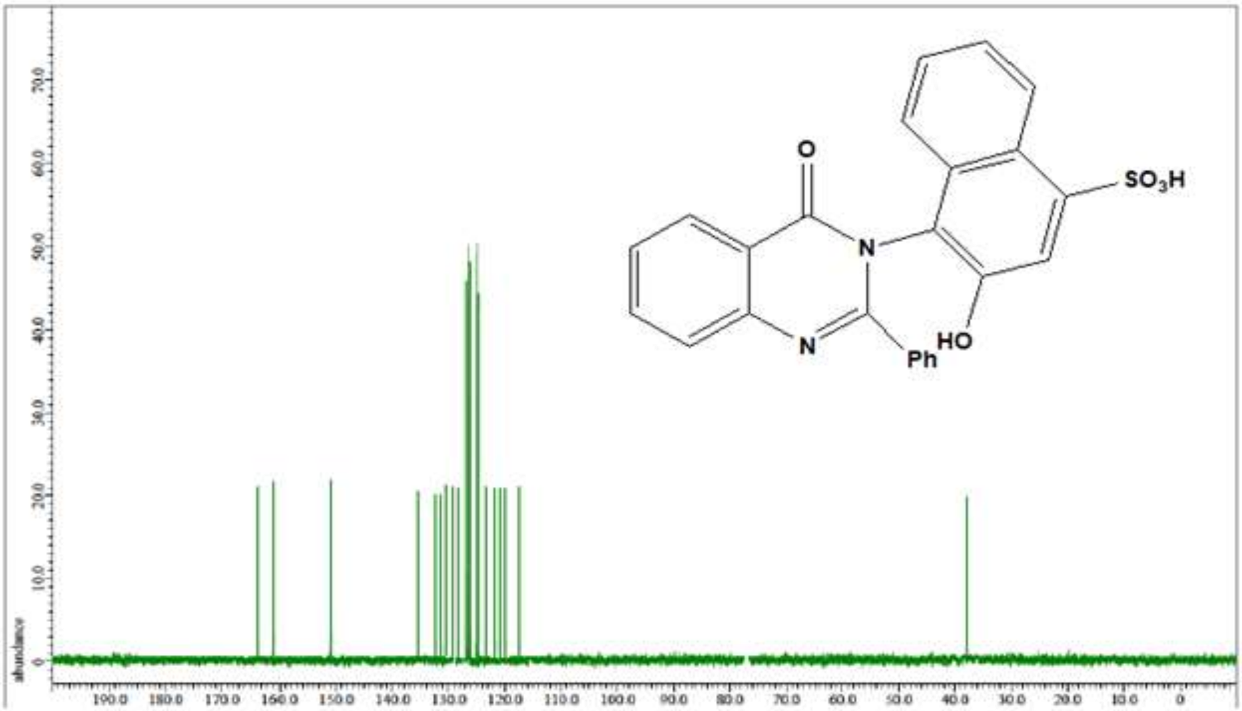

^{13}C-NMR spectrum of compound 11.

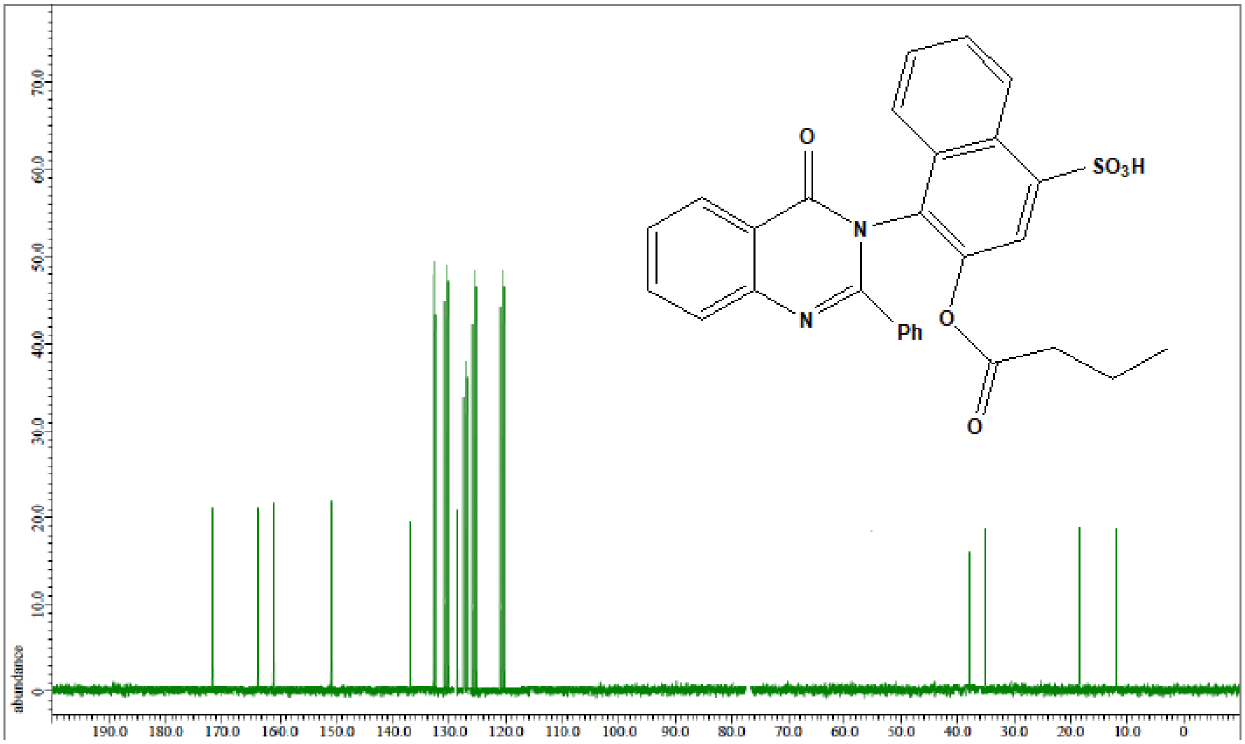

^{13}C-NMR spectrum of compound 12.

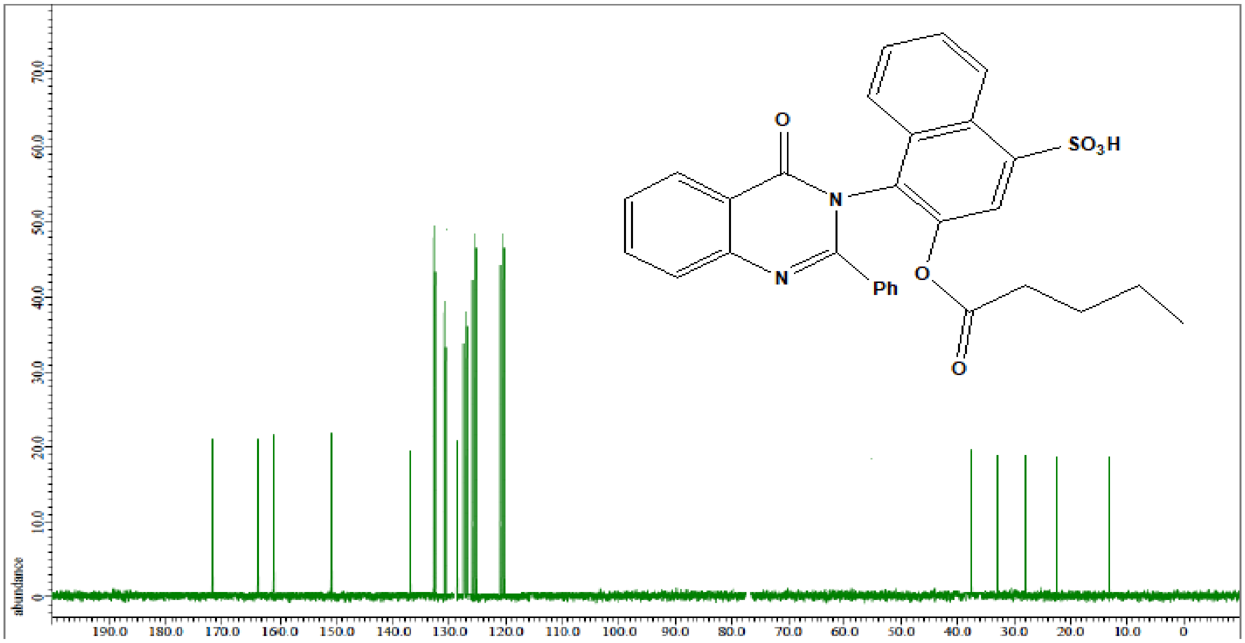

^{13}C-NMR spectrum of compound 13.

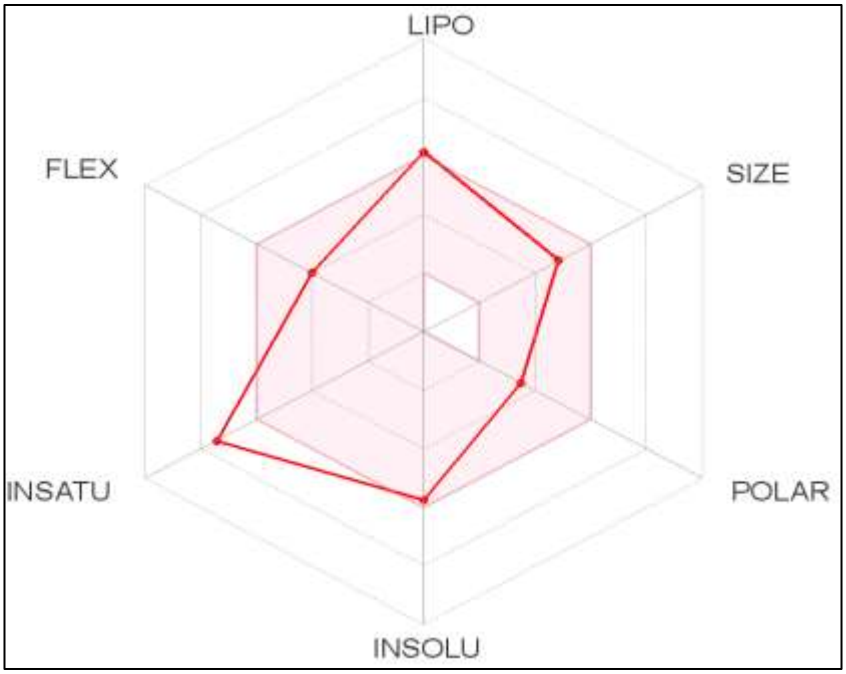

The oral bioavailability radar of the molecule 9. The colored zone is the suitable physicochemical space for oral bioavailability.

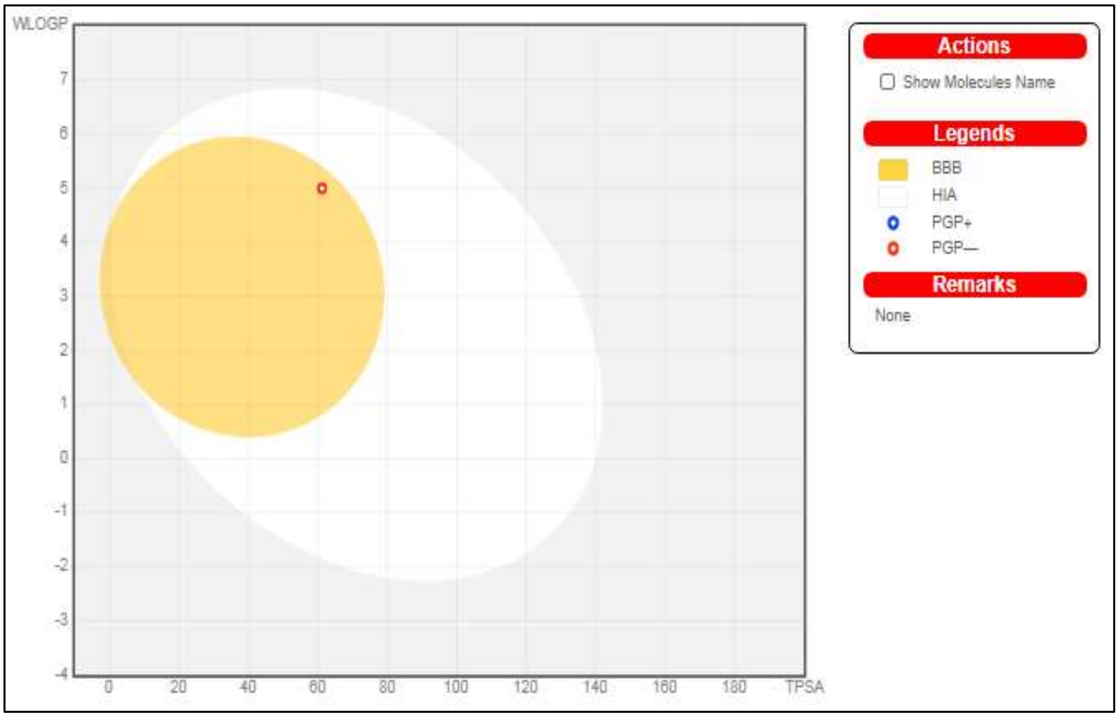

The Boiled-egg plot of the selected compound 9
